# Identification of an immune signature predicting prognosis risk of patients in lung adenocarcinoma

**DOI:** 10.1186/s12967-019-1824-4

**Published:** 2019-03-04

**Authors:** Qian Song, Jun Shang, Zuyi Yang, Lanlin Zhang, Chufan Zhang, Jianing Chen, Xianghua Wu

**Affiliations:** 1 Department of Medical Oncology, Fudan University Shanghai Cancer Center, No. 270 Dong-An Road, Shanghai, 200032 China; 2 Department of Oncology, Shanghai Medical College, Fudan University, 130 Dong-An Road, Shanghai, 200032 China; 3 School of Life Sciences, Fudan University, Shanghai, 200032 China; 4 Department of Hematology, The First Affiliated Hospital of Soochow University, Shizijie Campus: NO. 188, Shizijie Road, Suzhou, 215006 China

**Keywords:** Lung adenocarcinoma, Signature, Prognosis, Immune related gene, Tumor immune microenvironment, Mutation burden

## Abstract

**Background:**

Lung cancer has become the most common cancer type and caused the most cancer deaths. Lung adenocarcinoma (LUAD) is one of the major type of lung cancer. This study aimed to establish a signature based on immune related genes that can predict patients’ OS for LUAD.

**Methods:**

The expression data of 976 LUAD patients from The Cancer Genome Atlas database (training set) and the Gene Expression Omnibus database (four testing sets) and 1534 immune related genes from the ImmPort database were used for generation and validation of the signature. The glmnet Cox proportional hazards model was used to find the best gene model and construct the signature. To assess the independently prognostic ability of the signature, the Kaplan–Meier survival analysis and Cox’s proportional hazards model were performed.

**Results:**

A gene model consisting of 30 immune related genes with the highest frequency after 1000 iterations was used as our signature. The signature demonstrated robust prognostic ability in both training set and testing set and could serve as an independent predictor for LUAD patients in all datasets except GSE31210. Besides, the signature could predict the overall survival (OS) of LUAD patients in different subgroups. And this signature was strongly associated with important clinicopathological factors like recurrence and TNM stage. More importantly, patients with high risk score presented high tumor mutation burden.

**Conclusions:**

This signature could predict prognosis and reflect the tumor immune microenvironment of LUAD patients, which can promote individualized treatment and provide potential novel targets for immunotherapy.

**Electronic supplementary material:**

The online version of this article (10.1186/s12967-019-1824-4) contains supplementary material, which is available to authorized users.

## Background

According to the latest cancer statistics released in 2018, lung cancer has become the most frequently diagnosed cancer type and the top-ranked reason for cancer death in the combined population of women and men world widely [1]. In the United States, there were approximately 234,030 new cases and 154,050 deaths in 2018 [2]. Lung cancer mainly has two subtypes, including non-small cell lung cancer (NSCLC) and small cell lung cancer. Adenocarcinoma (LUAD) and squamous cell carcinoma are two main types of NSCLC [3], of which LUAD is the most common type [4]. With the decreasing of smoking rates, lung cancer cases of never-smoker are increasing, most of which comprise LUAD. For those patients, molecularly targeted therapies considerably enhance their survival outcomes. Tyrosine kinase inhibitors (TKIs) targeting epidermal growth factor receptor (EGFR) have been observed as the first-line treatment method for advanced LUAD patients with sensitizing EGFR mutation [5]. ROS proto-oncogene 1 (ROS1) and anaplastic lymphoma kinase (ALK) gene rearrangements are other common oncogenes which are somatically activated for the targeted therapies of LUAD [6]. However, a large amount of advanced LUAD patients do not have targetable mutations. For these patients, antibodies against immune checkpoints like programmed death 1 (PD-1) and cytotoxic T lymphocyte-associated antigen-4 (CTLA-4) demonstrate established treatment activity and safety [7, 8]. This highlights the importance of tumor immune microenvironment (TIM) on the clinical outcomes of LUAD patients.

The TIM constitutes of a variety of immune cells with either immune promoter or immune suppressor ability. TIM is able to limit the accumulation of T cells to where cancer cells locate [9]. Studies focusing on the impact of immune suppression elements like tumor-associated macrophages and myeloid-derived suppressor cells on LUAD patients’ survival outcomes have achieved tremendous development [10–13]. However, there has been no signature that can systematically evaluate the TIM on the basis of immune-related genes and predict LUAD patients’ overall survival or response to immunotherapies. Zheng et al. [14] recently demonstrated a signature based on B7-CD28 family that can predict LUAD patients’ prognosis. Nevertheless, their investigations were limited to B7-CD28 family members, which may not represent the status of the entire TIM. Therefore, it’s essential to develop an immune signature on the basis of a comprehensive list of immune-related genes that can stand for the immune status of TIM and be with prognostic ability in LUAD.

Our efforts concentrated on developing an immune signature with prognostic ability based on the comprehensive list of immune-related genes downloaded from The Immunology Database and Analysis Portal (ImmPort) database. The RNA sequencing (RNA-seq) data and microarray data from The Cancer Genome Atlas (TCGA) database and the Gene Expression Omnibus (GEO) database were used for analysis. Then, we evaluated whether this signature was associated with the survival outcome of subgroups of LUAD patients and clinicopathological factors. And finally, we tried to figure out the relationship between the signature and tumor immune-related indexes including mutation load and neoantigen in LUAD.

## Methods

### Publicly attainable expression datasets and immune related genes

The expression data were downloaded from the TCGA database and the GEO database. The RNA-seq data of 500 LUAD patients were collected from the TCGA database and used as the training set, which were downloaded from University of California Santa Cruz (UCSC) Genome Browser (https://xena.ucsc.edu/public-hubs/). GSE 81089 was the other RNA-seq data of 108 LUAD patients downloaded from the GEO database (http://www.ncbi.nlm.nih.gov/geo), which was used as one of the testing sets for constructing this signature. Fragments per kilobase of exon per million fragments mapped (FPKM) value was used to measure all of the RNA-seq data. The microarray data from GSE30219 (N = 85), GSE31210 (N = 226), GSE3141 (N = 57) were also collected from the GEO database and used as testing sets, respectively. A total of 976 patients were included for analysis. The clinical and survival information of the included datasets were summarized in Table 1. The comprehensive list of immune related genes containing a total of 1534 genes were downloaded from the ImmPort database (https://immport.niaid.nih.gov) [15].

**Table 1:** Clinical characteristics of the included datasets

Features	TCGA (n, %)	GSE30219 (n, %)	GSE31210 (n, %)	GSE3141 (n, %)	GSE81089 (n, %)
Platform	Illumina HiSeq2000 RNA sequencing platform	Affymetrix Human Genome U133 Plus 2.0 Array	Affymetrix Human Genome U133 Plus 2.0 Array	Affymetrix Human Genome U133 Plus 2.0 Array	Illumina HiSeq2000 RNA sequencing platform
Age					
≤ 60 y	157 (31.4%)	43 (50.6%)	108 (47.8%)	–	20 (18.5%)
> 60 y	333 (66.6%)	42 (49.4%)	118 (52.2%)	–	88 (81.5%)
NA	10 (2.0%)	0 (0.0%)	0 (0.0%)	–	0 (0.0%)
Gender					
Male	230 (46.0%)	66 (77.6%)	105 (46.5%)	–	39 (36.1%)
Female	270 (54.0%)	19 (22.4%)	121 (53.5%)	–	69 (63.9%)
NA	0 (0.0%)	0 (0.0%)	0 (0.0%)	–	0 (0.0%)
Recurrence					
Yes	155 (31.0%)	27 (31.8%)	64 (28.3%)	–	–
No	285 (57.0%)	58 (68.2%)	162 (71.7%)	–	–
NA	60 (12.0%)	0 (0.0%)	0 (0.0%)	–	–
AJCC stage					
Stage I	268 (53.6%)	–	168 (74.3%)	–	62 (57.4%)
Stage II	119 (23.8%)	–	58 (25.7%)	–	19 (17.6%)
Stage III	80 (16.0%)	–	0 (0.0%)	–	24 (22.2%)
Stage IV	25 (5.0%)	–	0 (0.0%)	–	3 (2.8%)
Stage X	8 (1.6%)	–	0 (0.0%)	–	0 (0.0%)
T stage					
T1	167 (33.4%)	71 (83.5%)	–	–	–
T2	267 (53.4%)	12 (14.1%)	–	–	–
T3	45 (9%)	2 (2.4%)	–	–	–
T4	18 (3.6%)	0 (0.0%)	–	–	–
TX	3 (0.6%)	0 (0.0%)	–	–	–
N stage					
N0	324 (64.8%)	82 (96.5%)	–	–	–
N1–3	165 (33.0%)	3 (3.5%)	–	–	–
NX	11 (2.2%)	0 (0.0%)	–	–	–
M stage					
M0	332 (66.4%)	85 (100%)	–	–	–
M1	24 (4.8%)	0 (100%)	–	–	–
MX	144 (28.8%)	0 (100%)	–	–	–
Survival status					
Alive	318 (63.6%)	40 (47.1%)	191 (84.5%)	26 (45.6%)	59 (54.6%)
Dead	182 (36.4%)	45 (52.9%)	35 (15.5%)	31 (54.4%)	49 (45.4%)

*y* years, *TCGA* The Cancer Genome Atlas

### Development and validation of the immune signature for LUAD

The cases from the TCGA database were used as the training set to develop the immune signature. Univariate analysis and logRank test were used to identify immune related genes with prognostic ability. For the genes with prognostic ability, Cox proportional hazards model (iteration = 1000) with an lasso penalty was used to find the best gene model utilizing a R package called “glmnet” [16]. The best gene model was used to establish the immune signature. Then, the concordance (c)-index proposed by Harrell et al. [17] was applied to validate the predictive ability of the signature in all of the five datasets, by using the “survcomp” R package [18]. The larger c-index indicated the more accurate predictive ability of the model.

### Survival analysis

The Kaplan–Meier (K–M) survival curves were generated to graphically demonstrate the overall survival (OS) of the high-risk group and low-risk group which were stratified by the immune signature. The univariate and multivariate analyses of survival were conducted for both the immune signature and clinicopathologic factors. The R package called “survival” was utilized to perform the survival analysis.

### Mutation load and neoantigen analysis

Mutation data that contained somatic variants were stored in Mutation Annotation Format (MAF) form and were downloaded from Genomic Data Commons (GDC) (https://portal.gdc.cancer.gov/).

Nonsynonymous mutations were used for our investigations, considering the uncertainty of functional consequences of synonymous mutations. And nonsynonymous mutations were potential sources of neoantigen epitopes. Nonsynonymous mutations included missense mutation, nonsense mutation, splice site mutation, frameshift mutation, and inframe mutation. The total number of nonsynonymous mutations were utilized as the mutation burden of LUAD patients to investigate the relationship between the immune signature and patients’ mutation load. The single nucleotide polymorphism (SNP) was also analyzed for its association with our signature. The number of neoantigens was cited from a published study [19] so as to figure out the correlation of the signature with the number of neoantigens in LUAD patients.

### Statistical analysis

Student’s t test was conducted to make statistical comparison. The “ggplot” R package was used to generate boxplots. “ComplexHeatmap” R package was applied to generate heatmaps [20]. Two-tailed p values less than 0.05 were thought to be statistically significant. All of our analyses were conducted using R software version 3.5.1 (https://www.r-project.org/).

## Results

### Construction of immune signature

**Fig. 1:**
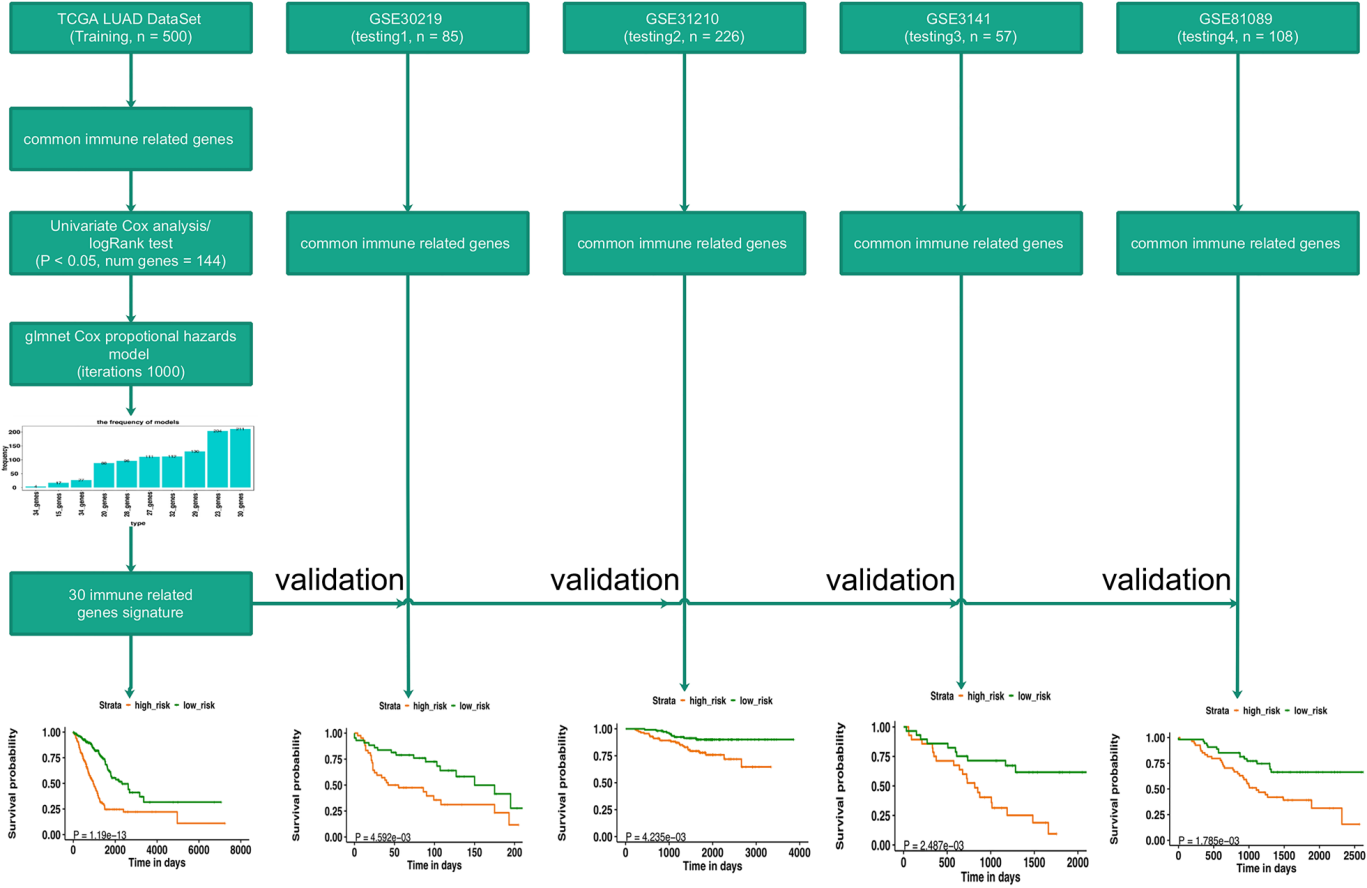
The workflow of construction and validation of the immune signature. The signature consisted of 30 immune related genes in LUAD which was constructed and validated using expression data from the TCGA database (training dataset) and the GEO database (four testing datasets). *TCGA* The Cancer Genome Atlas, *LUAD* lung adenocarcinoma

**Fig. 2:**
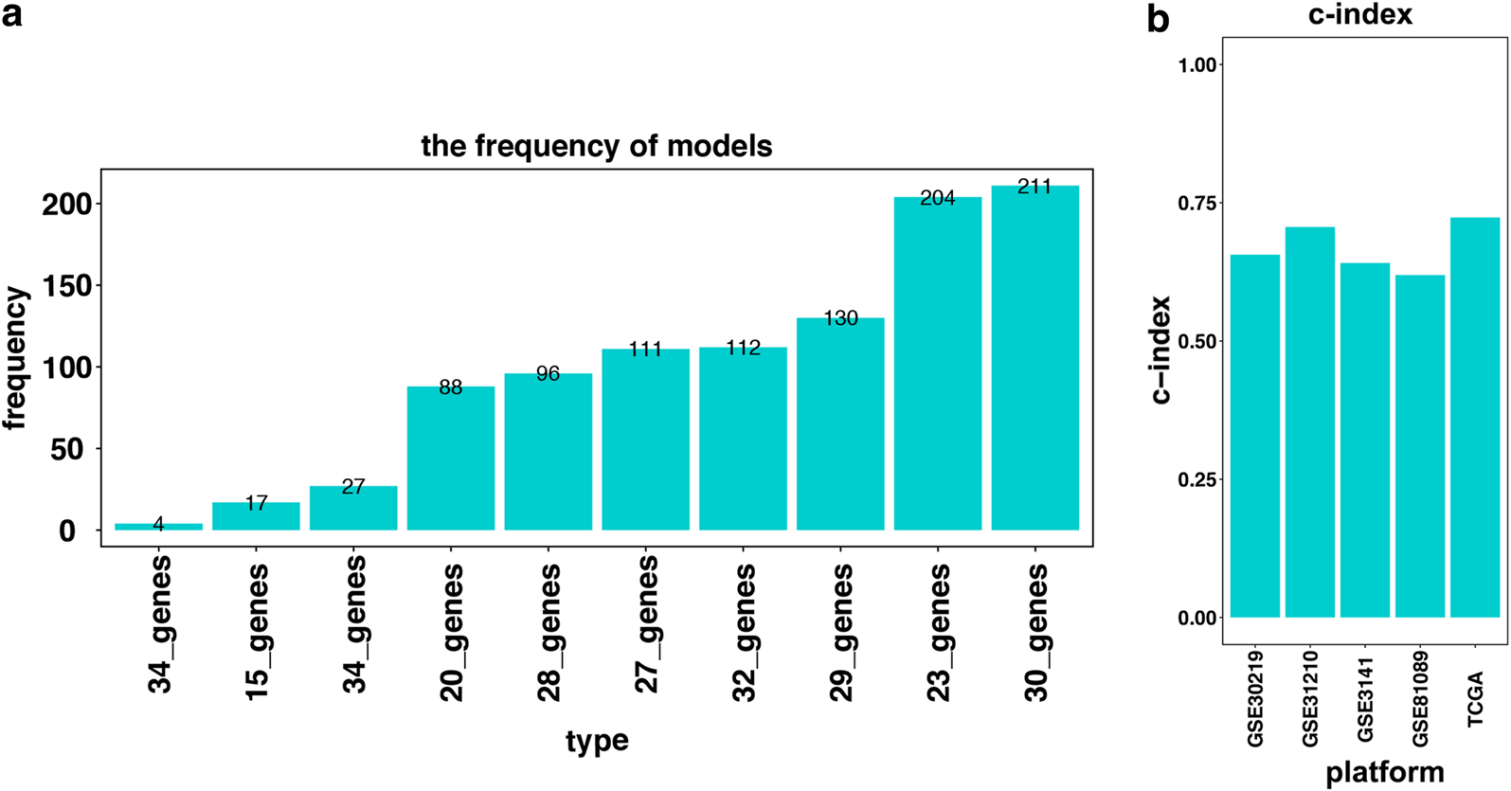
**a** Generation of the ten gene groups after 1000 iteration. The gene model with 30 immune related genes was selected to construct the signature as its highest frequencies of 211 compared to other nine gene models. **b** The c-index of both training and testing sets. The c-index for TCGA dataset, GSE30219, GSE31210, GSE3141, and GSE81089 were 0.723, 0.657, 0.7061, 0.641, and 0.619 respectively

To make our investigations clearer, a workflow that illustrated the generation of the signature was demonstrated in Fig. 1. The univariate analysis was performed in all of the 1534 immune related genes for TCGA LUAD datasets. There were 144 genes with prognostic ability after the univariate analysis and logRank test (P < 0.05). The 144 immune related genes then underwent the Cox proportional hazards regression with tenfold cross-validation to generate the best gene model. We totally performed 1000 iterations and included 10 gene groups for further screening. The gene lists of the 10 gene groups were shown in Additional file 1: Table S1. As illustrated in Fig. 2a, a gene model with 30 immune related genes was with the highest frequencies of 211 compared to other nine gene models. Thus, this gene model became the most suitable role to generate the immune signature for LUAD. Therefore, we utilized the 30 immune related genes in this gene model to construct our immune signature, as listed in Additional file 1: Table S1. The coefficient value of the 30 genes were listed in Additional file 2: Table S2. The prognostic ability of the 30 immune related genes in LUAD patients was confirmed in the training set (The TCGA dataset, Additional file 3: Figure S1), which showed that all of the 30 genes were able to predict survival outcome of LUAD patients. However, the prognostic ability of the 30 genes was not consistent in the four testing sets (The GEO datasets, Additional files 4, 5, 6, 7: Figures S2–S5).

### Validation of immune signature

**Fig. 3:**
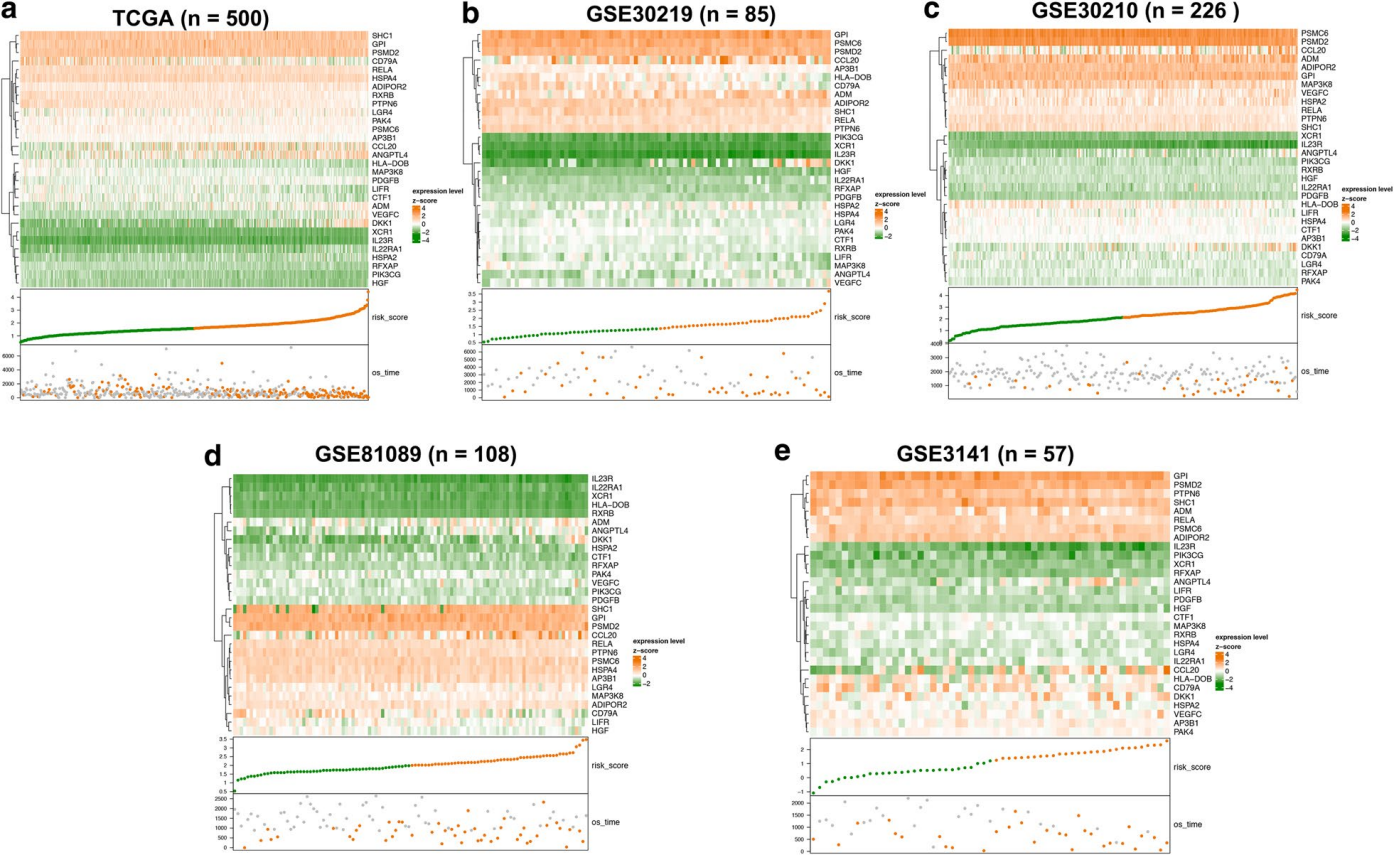
Heatmap of the signature consisting of 30 immune related genes in the TCGA dataset (**a**) and the GEO datasets, including GSE30219 (**b**), GSE30210 (**c**), GSE81089 (**d**), and GSE3141 (**e**). Patients were divided into high-risk and low-risk groups with the median risk score utilized as the cutoff value

**Fig. 4:**
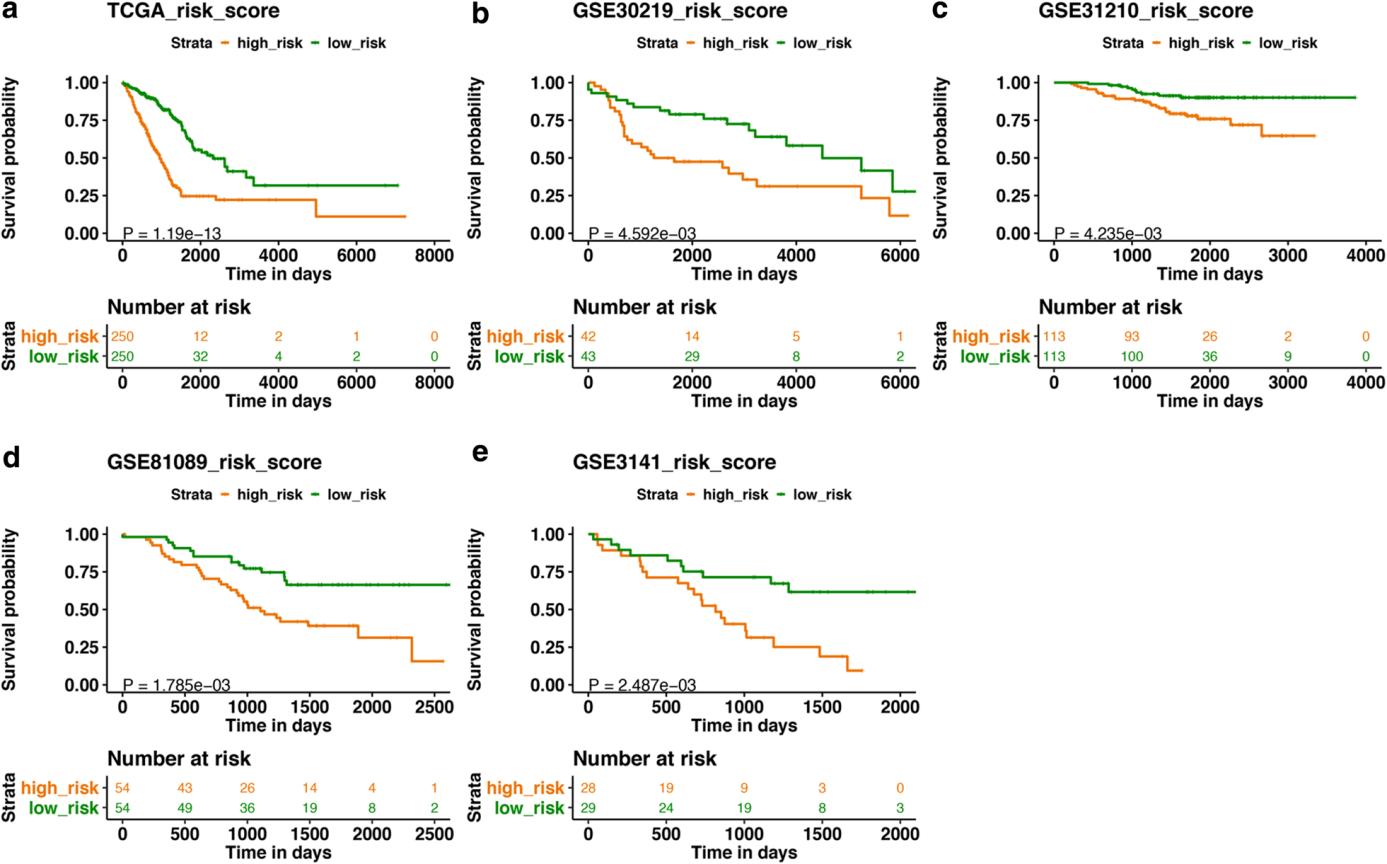
The Kaplan–Meier survival analysis of the signature for both training set and testing sets. Patients with high risk score demonstrated poor OS than those with low risk score in TCGA dataset (**a**), GSE30219 (**b**), GSE31210 (**c**), GSE81089 (**d**), and GSE3141 (**e**), which suggested the robust predictive ability for LUAD patients’ survival outcome (P < 0.001). *TCGA* The Cancer Genome Atlas

**Fig. 5:**
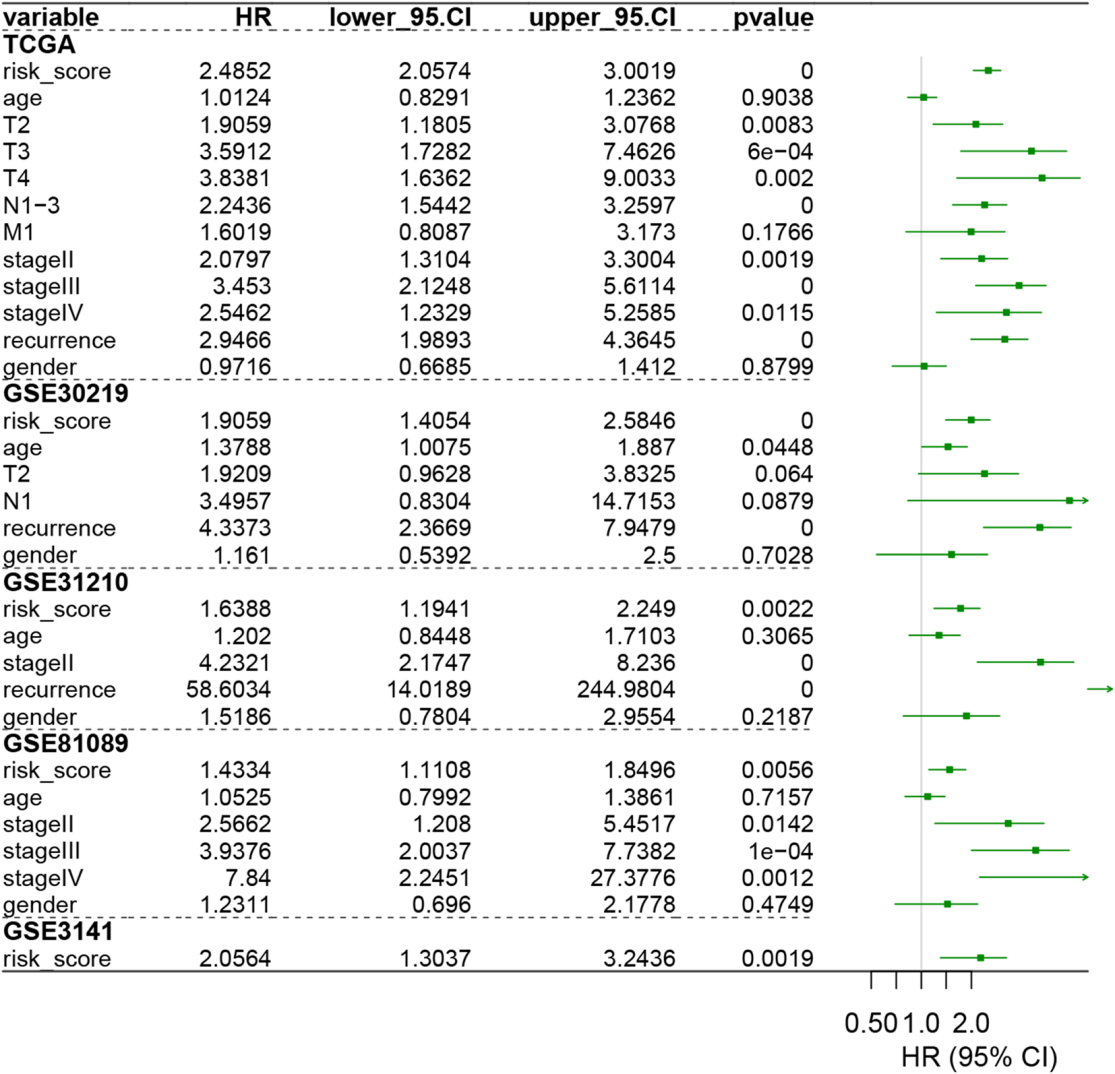
The Univariate Cox analysis of the signature and clinicopathological factors for training and testing sets. The HR in TCGA cohort was 2.4852 with 95% CI from 2.0574 to 3.0019 (P < 0.001). The HR in GSE30219 cohort was 1.9059 with 95% CI from 1.4054 to 2.5846 (P < 0.001). The HR in GSE31210 cohort was 1.6388 with 95% CI from 1.1941 to 2.249 (P = 0.0022). The HR in GSE81089 cohort was 1.4334 with 95% CI from 1.1108 to 1.8496 (P = 0.0056). The HR in GSE3141 cohort was 2.0564 with 95% CI from 1.3037 to 3.2436 (P = 0.0019). *TCGA* The Cancer Genome Atlas, *HR* hazard ratio, *95% CI* 95% confidence interval

**Fig. 6:**
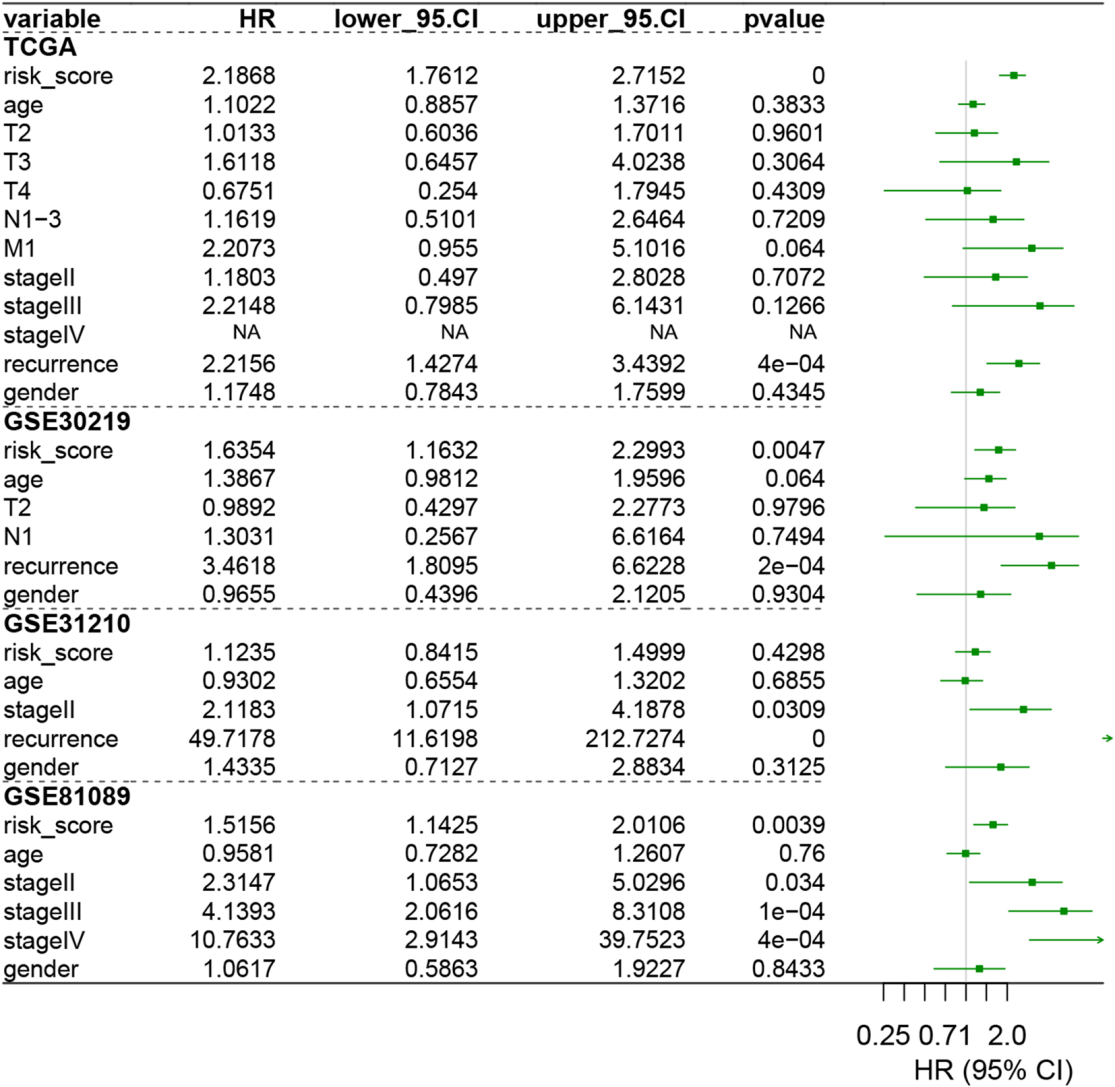
Multivariate Cox analysis evaluating independently predictive ability of our signature for OS. The signature was able to independently predict patients’ OS in TCGA cohort [Hazard ratio (HR) = 2.1868, 95% confidence intervals (95% CI) 1.7612 to 2.7152, P < 0.001], GSE30219 cohort (HR = 1.6354, 95% CI 1.1632–2.2993, P = 0.0047), and GSE81089 (HR = 1.5156, 95% CI 1.1425–2.0106, P = 0.0039). However, this relationship could not find in GSE31210 (P = 0.4298). *TCGA* The Cancer Genome Atlas, *HR* hazard ratio, *95% CI* 95% confidence interval

To validate our signature, we firstly calculated the c-index for the prediction of OS. The c-index for TCGA dataset, GSE30219, GSE31210, GSE3141, and GSE81089 were 0.723, 0.657, 0.7061, 0.641, and 0.619 respectively (P < 0.05, Fig. 2b), which indicated the high predictive accuracy of the signature for survival. Then, the risk score for each patient was calculated according to the coefficient value of the 30 genes. Patients were divided into high-risk and low-risk groups with the median risk score utilized as the cutoff value, as demonstrated in Fig. 3a–e. Patients of high-risk were with poor OS compared with those of low-risk in both TCGA and GEO datasets (Fig. 4a–e, P < 0.05). We further validated the prognostic ability of the signature in subgroups of LUAD, and we found the immune signature could also predict the survival outcome of patients in clinically important subgroups. In TCGA datasets, patients in high risk group demonstrated poor prognosis in T1–3 stage, N0–3 stage, M0–1 stage, stage I–IV, recurrence, and no recurrence (P < 0.05, Additional file 8: Figure S6). In GSE30219 datasets, patients in high risk group showed poor prognosis in T1 stage, N0 stage (P < 0.05, Additional file 9: Figure S7). In GSE31210 dataset, high risk patients in stage I group demonstrated poor survival outcome (P < 0.05, Additional file 10: Figure S8). In GSE81089 datasets, patients in stage III exhibited a negative correlation between the risk score and patients’ OS (P < 0.05, Additional file 11: Figure S9). The univariate Cox analysis of the immune signature also indicated the significant association of the signature with LUAD patients’ OS in both TCGA and GEO datasets (P < 0.05, Fig. 5). Multivariate Cox analysis further exhibited that our signature could serve as an independent predictor of patients’ survival outcome after adjusted by clinicopathologic factors including age, TNM stage, recurrence, and gender in TCGA cohort [Hazard ratio (HR) = 2.1868, 95% confidence intervals (95% CI) 1.7612 to 2.7152, P < 0.001], GSE30219 cohort (HR = 1.6354, 95% CI 1.1632–2.2993, P = 0.0047), and GSE81089 (HR = 1.5156, 95% CI 1.1425–2.0106, P = 0.0039), as demonstrated in Fig. 6. As for the prognostic ability of clinical factors, we found only tumor recurrence and stage IV could serve as independent predictors for patients’ OS, which indicated the strong prognostic ability of our signature.

### Association with clinicopathologic factors

To further validate the clinical value of the 30-genes immune signature, we evaluated the relationship between the signature and clinicopathologic factors. In TCGA cohort, patients of high-risk were tended to have advanced T stage, N stage, M stage, pathological stage and were under high risk of recurrence (P < 0.05, Additional file 12: Figure S10). In GSE30219 cohort, high-risk score was associated with higher T stage and N stage (P < 0.05, Additional file 13: Figure S11). In GSE31210 cohort, the risk score was only positively related to advanced pathological stage (P < 0.05, Additional file 14: Figure S12). And we did not find the association of the risk score and pathological stage in GSE81089 (Additional file 15: Figure S13).

### Association with mutation load and neoantigen

**Fig. 7:**
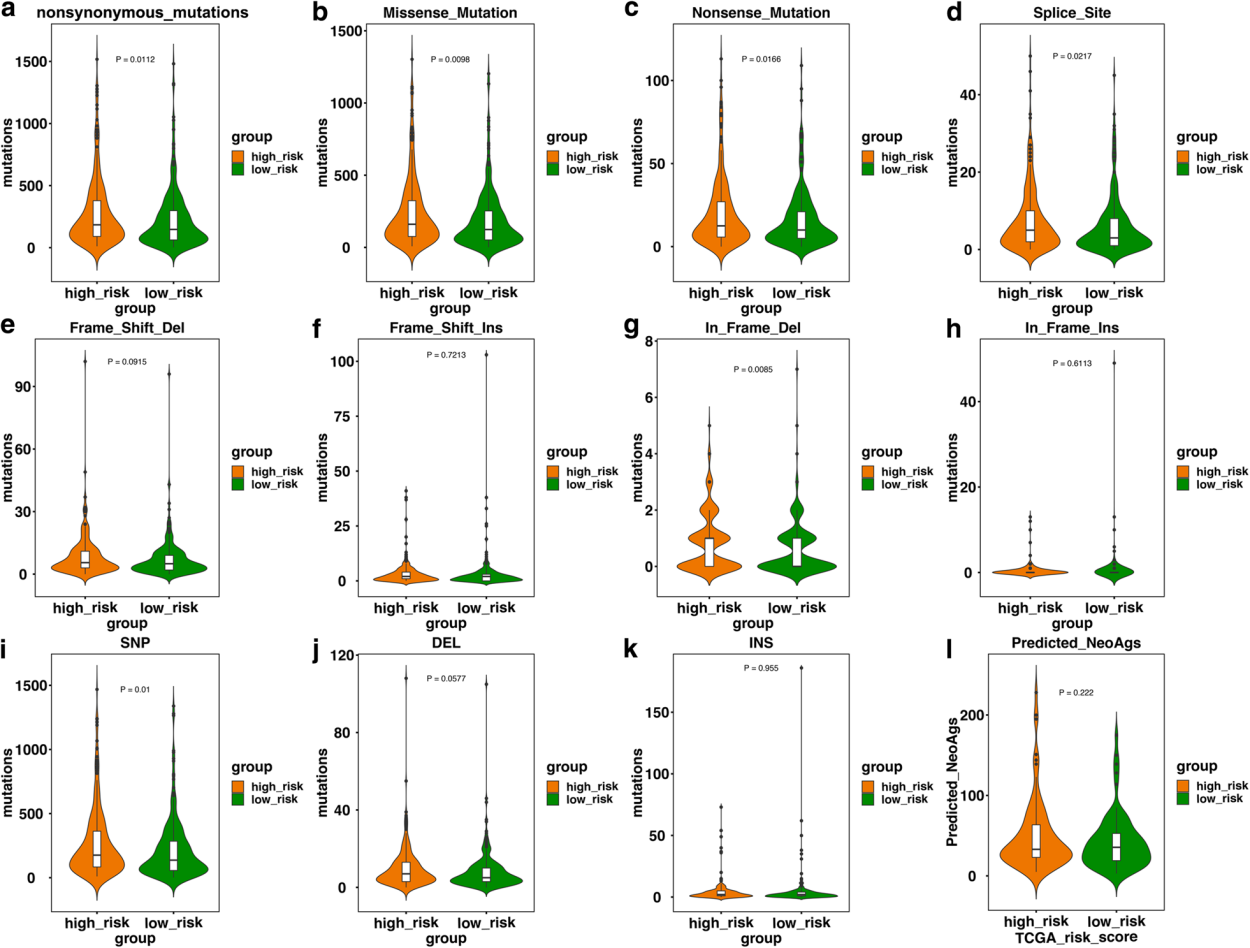
The relationship between the risk signature and nonsynonymous mutation burden, different nonsynonymous mutation types, and neoantigen. **a** High-risk group patients had higher nonsynonymous mutation burden (P = 0.0112). **b** Patients in high-risk group were associated with higher number of missense mutation (P = 0.0098). **c** Patients in high-risk group were associated with higher number of nonsense mutation (P = 0.0166). **d** Patients in high-risk group were associated with higher number of splice site (P = 0.0217). **e** There was no association of the risk score with the number of frame shift deletion. **f** There was no association of the risk score with the number of frame shift insertion. **g** Patients in high-risk group was associated with higher number of inframe deletion (P = 0.0085). **h** There was no association of the risk score with the number of inframe insertion. **i** Patients in high-risk group was associated with higher number of SNP (P = 0.01). **j** There was no association of the risk score with total number of deletion mutation. **k** There was no association of the risk score with total number of insertion mutation. **l** There was no association of the risk score with the number of neoantigen. *Del* deletion, *Ins* insertion, *SNP* single nucleotide polymorphism, *NeoAgs* neoantigens, *TCGA* The Cancer Genome Atlas

Higher nonsynonymous mutation burden load and neoantigen number have shown associations with clinical efficacy of immune checkpoint inhibitor therapy [21, 22]. Therefore, we would like to investigate whether our immune signature could affect mutation load and number of neoantigen of LUAD for the possibility of the risk score to be the predictor of response to immune checkpoint inhibitor. Patients with high risk score exhibited higher nonsynonymous mutation load than those with low risk score (P = 0.0112, Fig. 7a). To further explore which types of nonsynonymous mutation were the major contributors to this relationship, we evaluated the association of the signature with different types of nonsynonymous mutation. High-risk group patients had higher missense mutation (P = 0.0098, Fig. 7b), nonsense mutation (P = 0.0166, Fig. 7c), splice site mutation (P = 0.0217, Fig. 7d), and inframe deletion (P = 0.0085, Fig. 7g). We did not find this relationship in frameshift mutation (Fig. 7e, f), inframe insertion (Fig. 7h), total deletion mutation (Fig. 7j), and total insertion mutation (Fig. 7k). Besides, we found there demonstrated a positive correlation between the signature and the number of SNP (P = 0.0098, Fig. 7i). However, we did not find correlation between the signature and the number of neoantigens in LUAD (Fig. 7l).

## Discussion

The treatment of LUAD has experienced huge evolvement in the past 30 years, especially with the efficacy of immunotherapy. This shades light on the important role of TIM in the development and progression of LUAD. In this investigation, we established a robust prognostic signature on the basis of TIM in TCGA dataset and proved its efficacy in four GEO datasets. Our signature may represent the status of TIM for LUAD patients and provide potential biomarkers for the response to immunotherapy and targets for immunotherapy.

The study found that our signature was significantly correlated with LUAD patients’ OS. And their correlation showed a high statistical significance in the training set, testing sets, and the subgroups of LUAD patients, which indicates the signature is able to provide a robust prognostic tool for the total cohort and subgroups of LUAD patients. Besides, the signature showed strong correlation with clinicopathologic factors, further highlighting the firmly prognostic ability of our signature. In addition, we found that some of the 30 immune genes had different prognostic ability in different datasets. This indicates the instability of a single gene in predicting the OS of LUAD patients, while the signature which integrates the efficacy of all the 30 immune related genes showed a consistent predictive ability of OS in all of the datasets. Therefore, this immune signature has a greater value than a single gene in predicting patients’ survival outcome.

More importantly, our signature was on the basis of immune related genes and demonstrated a positive association with nonsynonymous mutation load and different types of nonsynonymous mutation. Considering the importance of tumor mutation load in predicting the response to immunotherapy [23], we could confer there may be a connection between our signature and response to immunotherapy. The NF-κB is a key participant in both immune response and human cancer initiation and progression [24–26]. Therefore, NF-κB is a crucial part linking immunity and cancer. Interestingly, there was a study demonstrating that inhibition of NF-κB c-Rel could impair regulatory T cells mediated immunosuppression and potentiate anti-PD-1 therapy efficacy [27]. Among the 30 immune related genes in our signature, RELA is a subunit of NF-κB that is essential for NF-κB activation [28]. Hence, this further indicates that our signature may be related to response to immunotherapy. Considering the neoantigen have also shown its ability to predict the response to immunotherapy in cancer patients, we investigated the relationship between the signature and the neoantigens. However, we did not find the correlation between the signature and number of neoantigens. Therefore, further validations are needed to evaluate this immune signature in LUAD patients treated with immune checkpoint blockade.

Nonetheless, there were several limitations in our investigation. First, the signature was developed using retrospective data. Therefore, clinical validation, or even gene expression data of the thirty genes in enough number of LUAD samples are needed to prove the efficacy of the signature. And we did not find the independently prognostic ability of the signature in all of the datasets, which may be caused by the diversity of different platforms, batch effects and limited number of samples. Besides, in some subgroups of LUAD patients, there was no correlation between the signature and OS, which was also caused by the limited sample number in subgroups. Finally, lacking in patients treated with immune checkpoint inhibitors, we are unable to confirm relationship between the signature and the response to immunotherapy.

## Conclusions

In conclusion, this study generates a signature that can not only predict LUAD patients’ survival outcome but also reflect the immune status of LUAD. This signature can be clinically used for the improvement of patients’ OS, individualized therapy methods based on the risk score and possible response to immunotherapy.

## Additional files

**Additional file 1: Table S1. ** The gene lists of the 10 gene groups.

**Additional file 2: Table S2.** The coefficient value of 30 immune related risk genes.

**Additional file 3: Figure S1.** The Kaplan–Meier survival analysis for the 30 immune related genes in TCGA dataset. The 30 immune related genes used to construct the immune signature demonstrated strong prognostic ability for LUAD patients’ OS in TCGA dataset (P < 0.001).

**Additional file 4: Figure S2.** The Kaplan–Meier survival analysis for the 30 immune related genes in GSE30219 dataset. Some of the 30 immune related genes used to construct the immune signature demonstrated strong prognostic ability for LUAD patients’ OS in GSE30219 dataset, while others did not exhibit prognostic ability.

**Additional file 5: Figure S3.** The Kaplan–Meier survival analysis for the 30 immune related genes in GSE31210 dataset. Some of the 30 immune related genes used to construct the immune signature demonstrated strong prognostic ability for LUAD patients’ OS in GSE31210 dataset, while others did not exhibit prognostic ability.

**Additional file 6: Figure S4.** The Kaplan–Meier survival analysis for the 30 immune related genes in GSE81089 dataset. Some of the 30 immune related genes used to construct the immune signature demonstrated strong prognostic ability for LUAD patients’ OS in GSE81089 dataset, while others did not exhibit prognostic ability.

**Additional file 7: Figure S5.** The Kaplan–Meier survival analysis for the 30 immune related genes in GSE3141 dataset. Some of the 30 immune related genes used to construct the immune signature demonstrated strong prognostic ability for LUAD patients’ OS in GSE3141 dataset, while others did not exhibit prognostic ability.

**Additional file 8: Figure S6.** The Kaplan–Meier survival analysis of the signature for LUAD subgroup patients in TCGA dataset. Patients of high-risk exhibited poor prognosis in T1 stage cohort, T2 stage cohort, T3 stage cohort, N0 stage cohort, N1–3 stage cohort, M0 stage cohort, M1 stage cohort, stage I cohort, stage II cohort, stage III cohort, stage IV cohort, recurrence cohort, and no recurrence cohort (P < 0.05). There was no association of the risk score with patients of T4 stage cohort. Abbreviations: The Cancer Genome Atlas (TCGA).

**Additional file 9: Figure S7.** The Kaplan–Meier survival analysis of the signature for LUAD subgroup patients in GSE30219 dataset. Patients of high-risk exhibited poor prognosis in T1 stage cohort and N0 stage cohort (P < 0.05). There was no association of the risk score with patients of T2 stage cohort, recurrence cohort, and no recurrence cohort.

**Additional file 10: Figure S8.** The Kaplan–Meier survival analysis of the signature for LUAD subgroup patients in GSE31210 dataset. Patients of high-risk exhibited poor prognosis in stage I cohort (P < 0.05). There was no association of the risk score with patients of stage II cohort, recurrence cohort, and no recurrence cohort.

**Additional file 11: Figure S9.** The Kaplan–Meier survival analysis of the signature for LUAD subgroup patients in GSE81089 dataset. Patients of high-risk exhibited poor prognosis in stage III cohort (P < 0.05). There was no association of the risk score with patients of stage I cohort, stage II cohort, and stage IV cohort.

**Additional file 12: Figure S10.** Correlation of the risk signature with clinicopathologic factors in TCGA datasets. The signature was positively correlated with T stage, N stage, M stage and pathologic stage in TCGA datasets (P < 0.05). Abbreviations: The Cancer Genome Atlas (TCGA).

**Additional file 13: Figure S11.** Correlation of the risk signature with clinicopathologic factors in GSE30219 datasets. The signature was positively correlated with T stage and N stage in GSE30219 datasets (P < 0.05). But there was no correlation of the signature and recurrence.

**Additional file 14: Figure S12.** Correlation of the risk signature with clinicopathologic factors in GSE31210 datasets. The signature was positively correlated with pathologic stage in GSE31210 datasets (P < 0.05). But there was no correlation of the signature and recurrence.

**Additional file 15: Figure S13.** Correlation of the risk signature with clinicopathologic factors in GSE81089 datasets. There was no correlation of the signature and pathologic stage in GSE81089, which may be caused by the small number of stage IV patients.

## Abbreviations

NSCLC: non-small cell lung cancer
LUAD: Lung Adenocarcinoma
TKIs: tyrosine kinase inhibitors
EGFR: epidermal growth factor receptor
ROS1: ROS proto-oncogene 1
ALK: anaplastic lymphoma kinase
PD-1: programmed death 1
CTLA-4: cytotoxic T lymphocyte-associated antigen-4
TIM: tumor immune microenvironment
ImmPort: The Immunology Database and Analysis Portal
RNA-seq: RNA sequencing
TCGA: The Cancer Genome Atlas
GEO: the Gene Expression Omnibus
UCSC: University of California Santa Cruz
c-index: concordance index
K–M: Kaplan–Meier
OS: overall survival
MAF: Mutation Annotation Format
GDC: Genomic Data Commons
SNP: single nucleotide polymorphism

## References

[1] Bray F, Ferlay J, Soerjomataram I, Siegel RL, Torre LA, Jemal A (2018). Global cancer statistics 2018: GLOBOCAN estimates of incidence and mortality worldwide for 36 cancers in 185 countries. CA Cancer J Clin.

[2] Siegel RL, Miller KD, Jemal A (2018). Cancer statistics, 2018. CA Cancer J Clin.

[3] Gridelli C, Rossi A, Carbone DP, Guarize J, Karachaliou N, Mok T (2015). Non-small-cell lung cancer. Nat Rev Dis Primers.

[4] Network CGAR (2014). Comprehensive molecular profiling of lung adenocarcinoma. Nature.

[5] Zhou C, Di Yao L (2016). Strategies to improve outcomes of patients with EGFR-mutant non–small cell lung cancer: review of the literature. J Thoracic Oncol.

[6] Hanna N, Johnson D, Temin S, Baker S, Brahmer J, Ellis PM (2017). Systemic therapy for stage IV non-small-cell lung cancer: American Society of Clinical Oncology clinical practice guideline update. J Clin Oncol.

[7] Hellmann MD, Rizvi NA, Goldman JW, Gettinger SN, Borghaei H, Brahmer JR (2017). Nivolumab plus ipilimumab as first-line treatment for advanced non-small-cell lung cancer (CheckMate 012): results of an open-label, phase 1, multicohort study. Lancet Oncol.

[8] Xu X, Huang Z, Zheng L, Fan Y (2018). The efficacy and safety of anti-PD-1/PD-L 1 antibodies combined with chemotherapy or CTLA 4 antibody as a first-line treatment for advanced lung cancer. Int J Cancer.

[9] Joyce JA, Fearon DT (2015). T cell exclusion, immune privilege, and the tumor microenvironment. Science.

[10] Lesokhin AM, Hohl TM, Kitano S, Cortez C, Hirschhorn-Cymerman D, Avogadri F (2012). Monocytic CCR2 + myeloid-derived suppressor cells promote immune escape by limiting activated CD8 T-cell infiltration into the tumor microenvironment. Cancer research..

[11] Bayne LJ, Beatty GL, Jhala N, Clark CE, Rhim AD, Stanger BZ (2012). Tumor-derived granulocyte-macrophage colony-stimulating factor regulates myeloid inflammation and T cell immunity in pancreatic cancer. Cancer Cell.

[12] Strachan DC, Ruffell B, Oei Y, Bissell MJ, Coussens LM, Pryer N (2013). CSF1R inhibition delays cervical and mammary tumor growth in murine models by attenuating the turnover of tumor-associated macrophages and enhancing infiltration by CD8+ T cells. Oncoimmunology.

[13] Zhu Y, Knolhoff BL, Meyer MA, Nywening TM, West BL, Luo J (2014). CSF1/CSF1R blockade reprograms tumor-infiltrating macrophages and improves response to T cell checkpoint immunotherapy in pancreatic cancer models. Cancer Res.

[14] Zheng S, Luo X, Dong C, Zheng D, Xie J, Zhuge L (2018). A B7-CD28 family based signature demonstrates significantly different prognoses and tumor immune landscapes in lung adenocarcinoma. Int J Cancer.

[15] Bhattacharya S, Andorf S, Gomes L, Dunn P, Schaefer H, Pontius J (2014). ImmPort: disseminating data to the public for the future of immunology. Immunol Res.

[16] Friedman J, Hastie T, Tibshirani R (2010). Regularization paths for generalized linear models via coordinate descent. J Stat Softw.

[17] Harrell FE, Lee KL, Mark DB (1996). Multivariable prognostic models: issues in developing models, evaluating assumptions and adequacy, and measuring and reducing errors. Stat Med.

[18] Schröder MS, Culhane AC, Quackenbush J, Haibe-Kains B (2011). survcomp: an R/Bioconductor package for performance assessment and comparison of survival models. Bioinformatics.

[19] Rooney MS, Shukla SA, Wu CJ, Getz G, Hacohen N (2015). Molecular and genetic properties of tumors associated with local immune cytolytic activity. Cell.

[20] Gu Z, Eils R, Schlesner M (2016). Complex heatmaps reveal patterns and correlations in multidimensional genomic data. Bioinformatics.

[21] Rizvi NA, Hellmann MD, Snyder A, Kvistborg P, Makarov V, Havel JJ (2015). Mutational landscape determines sensitivity to PD-1 blockade in non–small cell lung cancer. Science.

[22] Gibney GT, Weiner LM, Atkins MB (2016). Predictive biomarkers for checkpoint inhibitor-based immunotherapy. Lancet Oncol.

[23] Goodman AM, Kato S, Bazhenova L, Patel SP, Frampton GM, Miller V (2017). Tumor mutational burden as an independent predictor of response to immunotherapy in diverse cancers. Mol Cancer Ther.

[24] Ghosh S, May MJ, Kopp EB (1998). NF-κB and Rel proteins: evolutionarily conserved mediators of immune responses. Annu Rev Immunol.

[25] Karin M, Cao Y, Greten FR, Li Z-W (2002). NF-κB in cancer: from innocent bystander to major culprit. Nat Rev Cancer.

[26] Karin M, Greten FR (2005). NF-κB: linking inflammation and immunity to cancer development and progression. Nat Rev Immunol.

[27] Grinberg-Bleyer Y, Oh H, Desrichard A, Bhatt DM, Caron R, Chan TA (2017). NF-κB c-Rel is crucial for the regulatory T cell immune checkpoint in cancer. Cell.

[28] Ghosh S, Karin M (2002). Missing pieces in the NF-κB puzzle. Cell.

