# Supplementary material for: Identification of an immune signature predicting prognosis risk of patients in lung adenocarcinoma
Source: J Transl Med. 2019 Mar 4;17:70. doi: 10.1186/s12967-019-1824-4 (PMC6399972; doi:10.1186/s12967-019-1824-4)
Supplement: Supplementary file 1 — Additional file 1: Table S1. The gene lists of the 10 gene groups. [file 12967_2019_1824_MOESM1_ESM.docx]

| **type** | **genes** | **frequency** |
| --- | --- | --- |
| 30_genes | PSMC6;LIFR;PIK3CG;CTF1;RELA;MAP3K8;HLA-DOB;LGR4;RXRB;CD79A;ADIPOR2;CCL20;PTPN6;HSPA4;GPI;ADM;IL22RA1;ANGPTL4;XCR1;AP3B1;RFXAP;HSPA2;IL23R;PDGFB;DKK1;PAK4;PSMD2;VEGFC;SHC1;HGF | 211 |
| 32_genes | PSMC6;LIFR;PIK3CG;CTF1;RELA;MAP3K8;LGR4;RXRB;CD79A;ADIPOR2;CCL20;PTPN6;MC1R;HSPA4;GPI;ADM;IL22RA1;ANGPTL4;CD1B;XCR1;AP3B1;RFXAP;HSPA2;IL23R;PDGFB;DKK1;PAK4;TNFRSF10C;PSMD2;VEGFC;SHC1;HGF | 112 |
| 15_genes | PIK3CG;MAP3K8;HLA-DOB;CD79A;CCL20;PTPN6;HSPA4;GPI;ADM;ANGPTL4;HSPA2;DKK1;PSMD2;VEGFC;SHC1 | 17 |
| 20_genes | PIK3CG;CTF1;MAP3K8;HLA-DOB;LGR4;CD79A;ADIPOR2;CCL20;PTPN6;HSPA4;GPI;ADM;IL22RA1;ANGPTL4;XCR1;HSPA2;DKK1;PSMD2;VEGFC;SHC1 | 88 |
| 28_genes | PSMC6;LIFR;PIK3CG;CTF1;MAP3K8;HLA-DOB;LGR4;RXRB;CD79A;ADIPOR2;CCL20;PTPN6;HSPA4;GPI;ADM;IL22RA1;ANGPTL4;XCR1;AP3B1;RFXAP;HSPA2;IL23R;PDGFB;DKK1;PSMD2;VEGFC;SHC1;HGF | 96 |
| 29_genes | PSMC6;LIFR;PIK3CG;CTF1;RELA;MAP3K8;HLA-DOB;LGR4;RXRB;CD79A;ADIPOR2;CCL20;PTPN6;HSPA4;GPI;ADM;IL22RA1;ANGPTL4;XCR1;AP3B1;RFXAP;HSPA2;IL23R;PDGFB;DKK1;PSMD2;VEGFC;SHC1;HGF | 130 |
| 23_genes | PIK3CG;CTF1;MAP3K8;HLA-DOB;LGR4;RXRB;CD79A;ADIPOR2;CCL20;PTPN6;HSPA4;GPI;ADM;IL22RA1;ANGPTL4;XCR1;HSPA2;PDGFB;DKK1;PSMD2;VEGFC;SHC1;HGF | 204 |
| 27_genes | PSMC6;LIFR;PIK3CG;CTF1;MAP3K8;HLA-DOB;LGR4;RXRB;CD79A;ADIPOR2;CCL20;PTPN6;HSPA4;GPI;ADM;IL22RA1;ANGPTL4;XCR1;RFXAP;HSPA2;IL23R;PDGFB;DKK1;PSMD2;VEGFC;SHC1;HGF | 111 |
| 34_genes | PSMC6;LIFR;PIK3CG;CTF1;RELA;MAP3K8;LGR4;RXRB;CD79A;ADIPOR2;CCL20;PTPN6;MC1R;HSPA4;GPI;ADM;IL22RA1;ANGPTL4;CD1B;XCR1;AP3B1;RFXAP;HSPA2;IL23R;PDGFB;PPP3CC;DKK1;PAK4;TNFRSF10C;PSMD2;VEGFC;IL6R;SHC1;HGF | 27 |
| 34_genes | PSMC6;LIFR;PIK3CG;CTF1;RELA;MAP3K8;LGR4;RXRB;CD79A;ADIPOR2;CCL20;PTPN6;MC1R;HSPA4;GPI;ADM;IL22RA1;ANGPTL4;CD1B;XCR1;AP3B1;RFXAP;HSPA2;IL23R;PDGFB;PPP3CC;DKK1;NRAS;PAK4;TNFRSF10C;VEGFC;IL6R;SHC1;HGF | 4 |

TableS1:
