# Supplementary material for: Identification of an immune signature predicting prognosis risk of patients in lung adenocarcinoma
Source: J Transl Med. 2019 Mar 4;17:70. doi: 10.1186/s12967-019-1824-4 (PMC6399972; doi:10.1186/s12967-019-1824-4)
Supplement: Supplementary file 2 — Additional file 2: Table S2. The coefficient value of 30 immune related risk genes. [file 12967_2019_1824_MOESM2_ESM.docx]

| **risk_genes** | **coefficient_value** | **risk_genes** | **coefficient_value** |
| --- | --- | --- | --- |
| PSMC6 | 0.109443821 | ADM | 0.040873151 |
| LIFR | -0.024850941 | IL22RA1 | 0.045088368 |
| PIK3CG | -0.044670589 | ANGPTL4 | 0.020599813 |
| CTF1 | -0.014488618 | XCR1 | -0.046012507 |
| RELA | 0.023063003 | AP3B1 | 0.082428691 |
| MAP3K8 | -0.197151664 | RFXAP | -0.023424644 |
| HLA-DOB | -0.002478115 | HSPA2 | 0.060920324 |
| LGR4 | 0.020143162 | IL23R | -0.697851531 |
| RXRB | -0.07784849 | PDGFB | 0.073486046 |
| CD79A | -0.075733088 | DKK1 | 0.10658978 |
| ADIPOR2 | 0.031812641 | PAK4 | 0.004893746 |
| CCL20 | 0.103954593 | PSMD2 | 0.024641501 |
| PTPN6 | -0.145440789 | VEGFC | 0.116668116 |
| HSPA4 | 0.108855641 | SHC1 | 0.089824064 |
| GPI | 0.057002004 | HGF | -0.045440712 |

Table S2: The coefficient value of 30 immune related risk genes
