# Supplementary figures and images for: Identification of an immune signature predicting prognosis risk of patients in lung adenocarcinoma
Source: J Transl Med. 2019 Mar 4;17:70. doi: 10.1186/s12967-019-1824-4 (PMC6399972; doi:10.1186/s12967-019-1824-4)

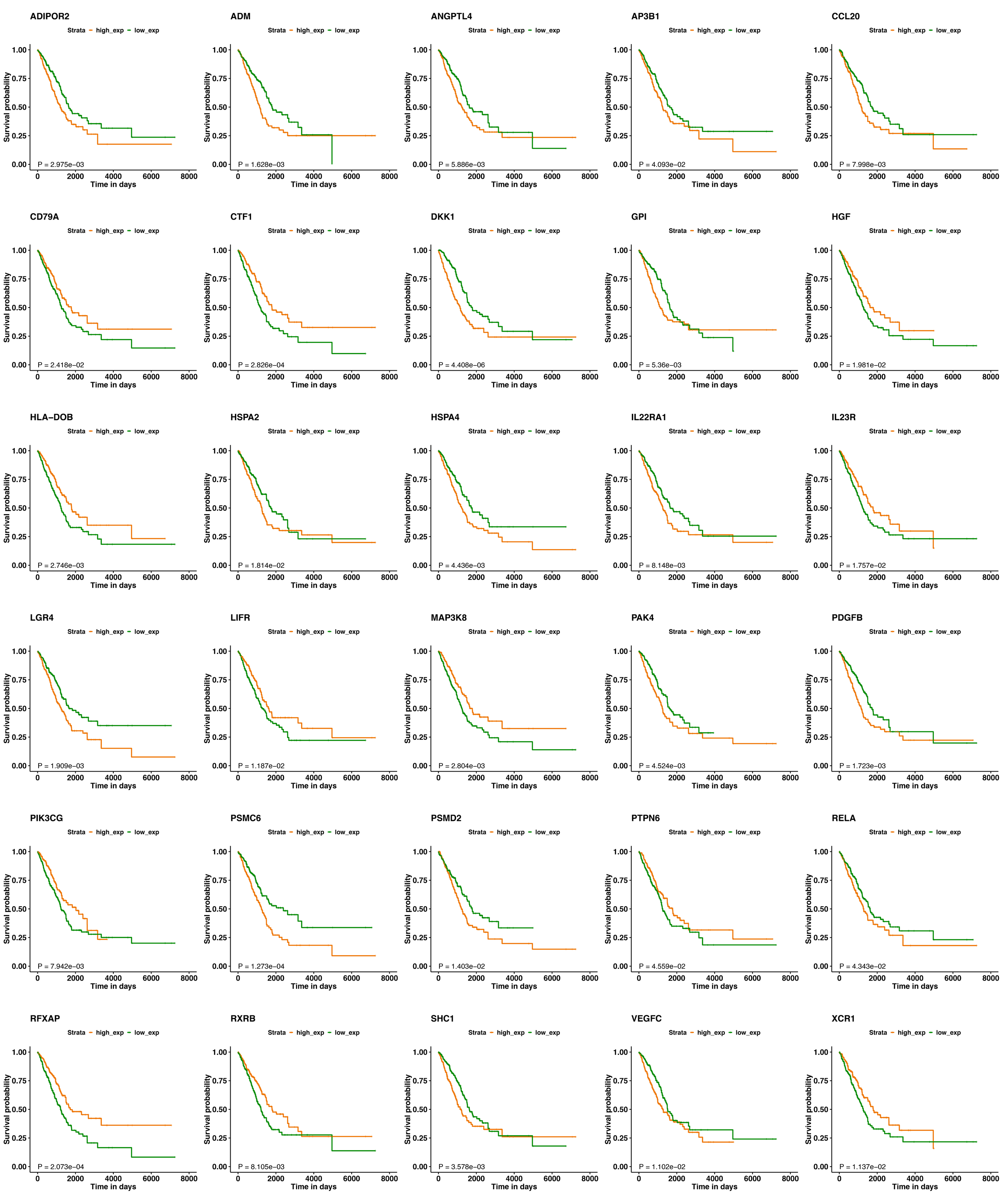

Supplement: Supplementary file 3 — Additional file 3: Figure S1. The Kaplan–Meier survival analysis for the 30 immune related genes in TCGA dataset. The 30 immune related genes used to construct the immune signature demonstrated strong prognostic ability for LUAD patients’ OS in TCGA dataset (P < 0.001). [file 12967_2019_1824_MOESM3_ESM.tif]

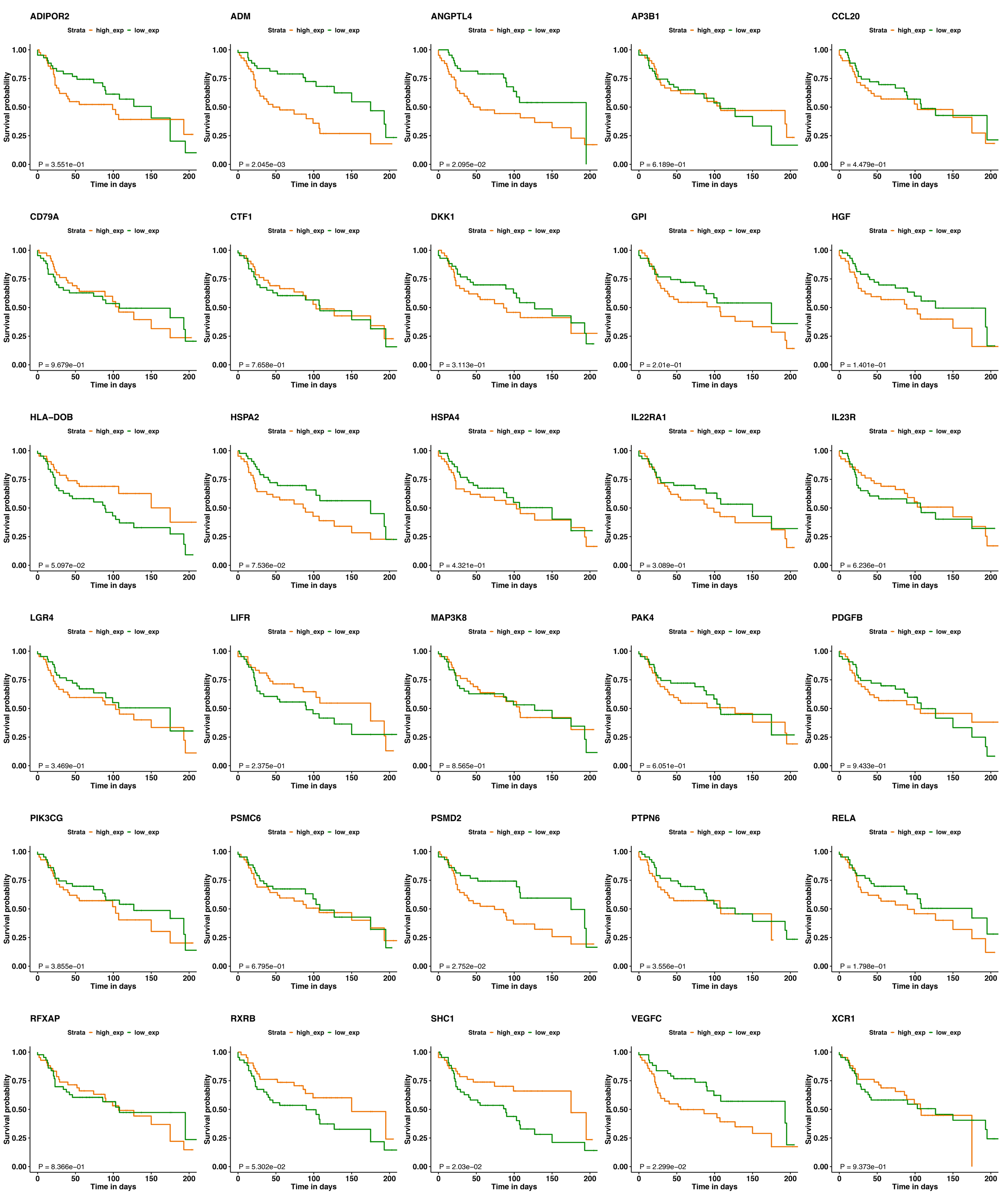

Supplement: Supplementary file 4 — Additional file 4: Figure S2. The Kaplan–Meier survival analysis for the 30 immune related genes in GSE30219 dataset. Some of the 30 immune related genes used to construct the immune signature demonstrated strong prognostic ability for LUAD patients’ OS in GSE30219 dataset, while others did not exhibit prognostic ability. [file 12967_2019_1824_MOESM4_ESM.tif]

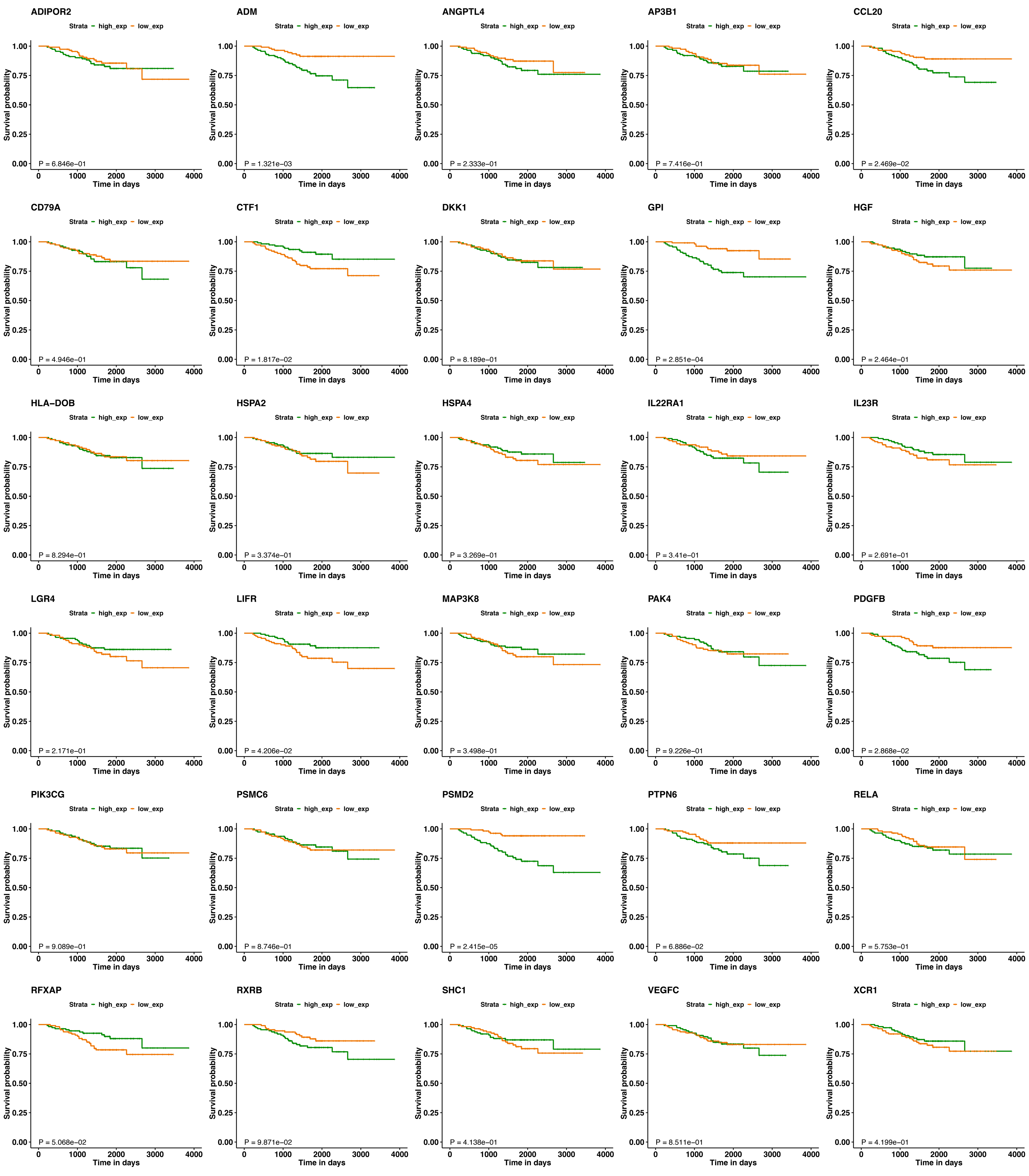

Supplement: Supplementary file 5 — Additional file 5: Figure S3. The Kaplan–Meier survival analysis for the 30 immune related genes in GSE31210 dataset. Some of the 30 immune related genes used to construct the immune signature demonstrated strong prognostic ability for LUAD patients’ OS in GSE31210 dataset, while others did not exhibit prognostic ability. [file 12967_2019_1824_MOESM5_ESM.tif]

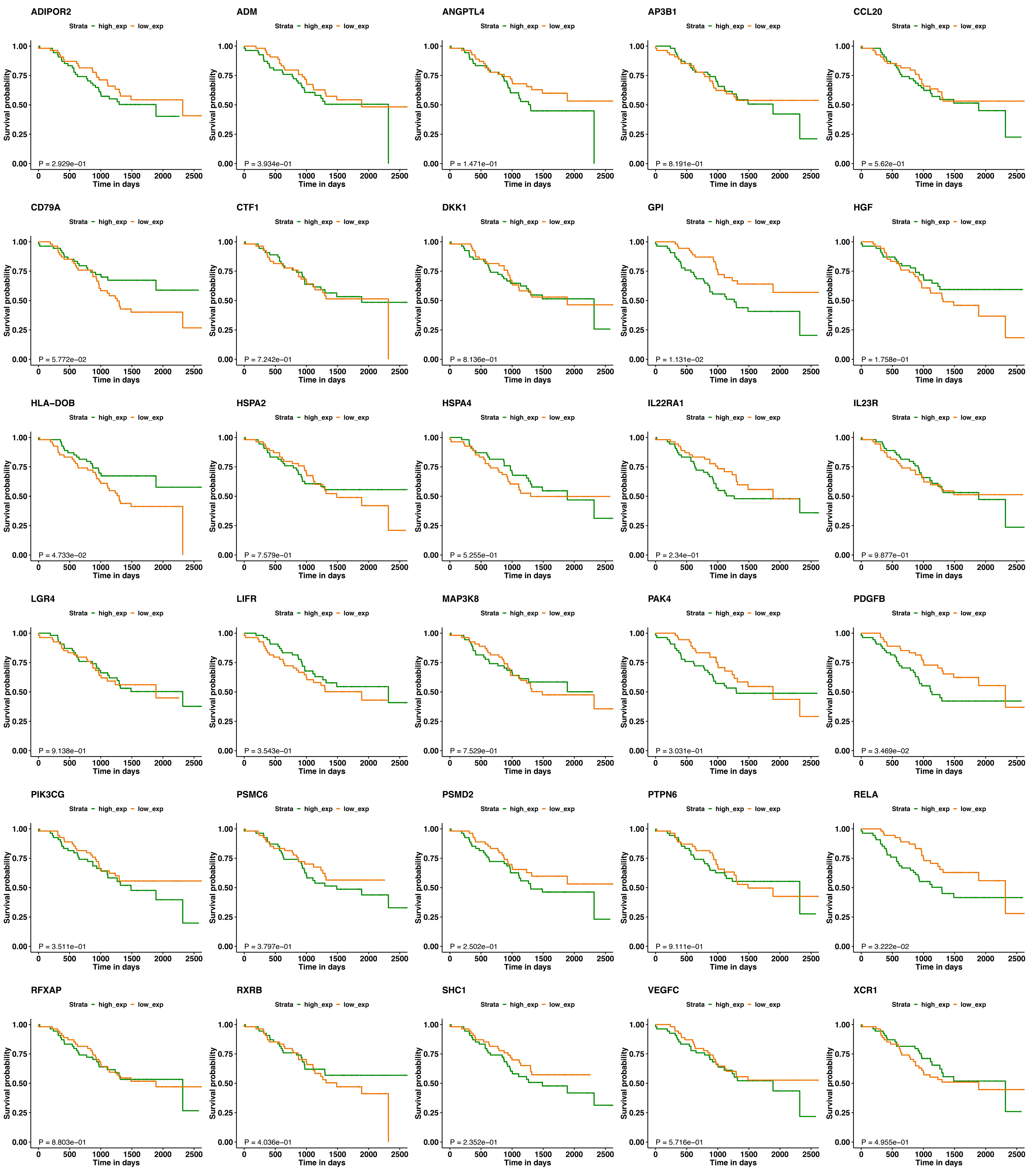

Supplement: Supplementary file 6 — Additional file 6: Figure S4. The Kaplan–Meier survival analysis for the 30 immune related genes in GSE81089 dataset. Some of the 30 immune related genes used to construct the immune signature demonstrated strong prognostic ability for LUAD patients’ OS in GSE81089 dataset, while others did not exhibit prognostic ability. [file 12967_2019_1824_MOESM6_ESM.tif]

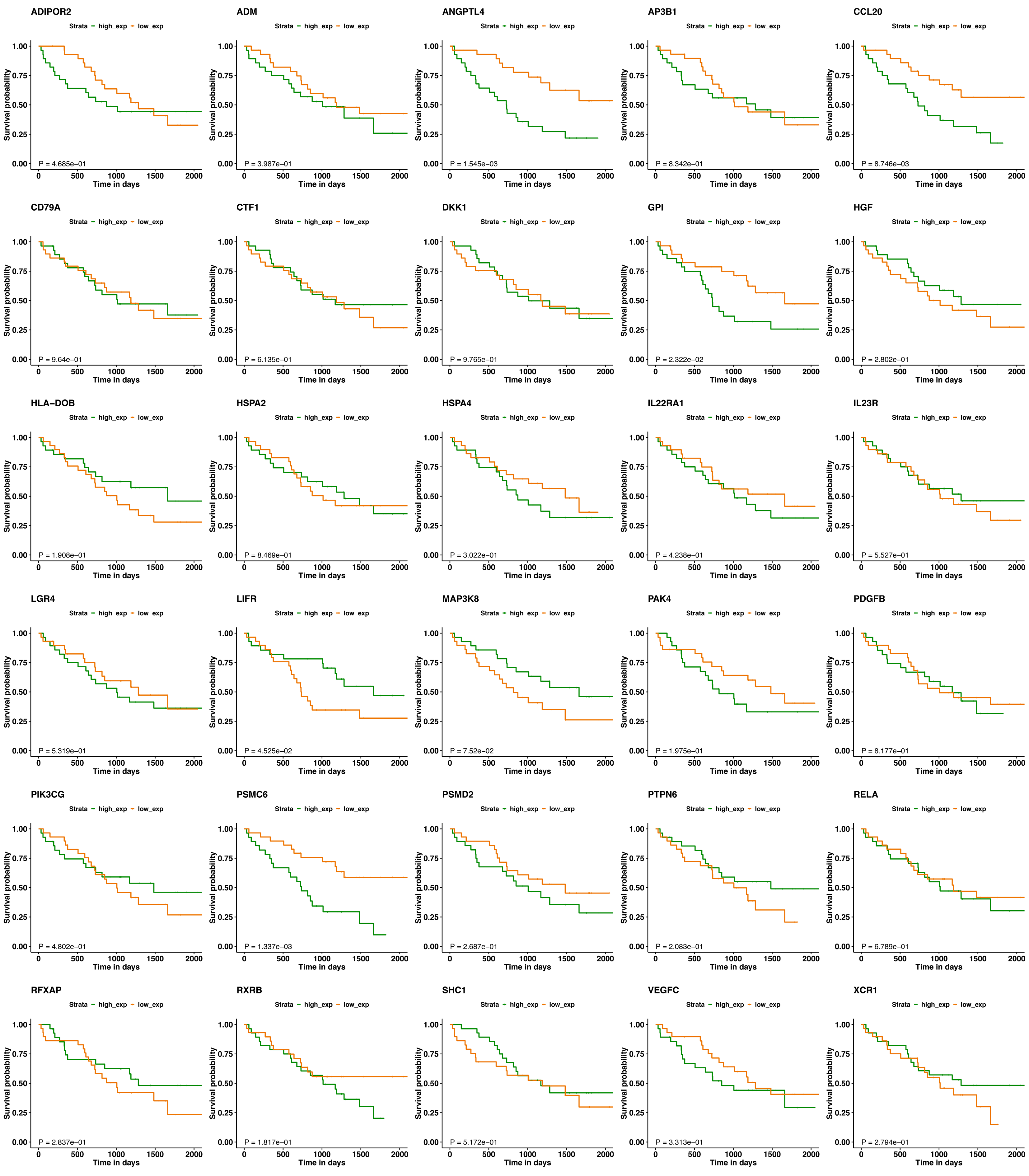

Supplement: Supplementary file 7 — Additional file 7: Figure S5. The Kaplan–Meier survival analysis for the 30 immune related genes in GSE3141 dataset. Some of the 30 immune related genes used to construct the immune signature demonstrated strong prognostic ability for LUAD patients’ OS in GSE3141 dataset, while others did not exhibit prognostic ability. [file 12967_2019_1824_MOESM7_ESM.tif]

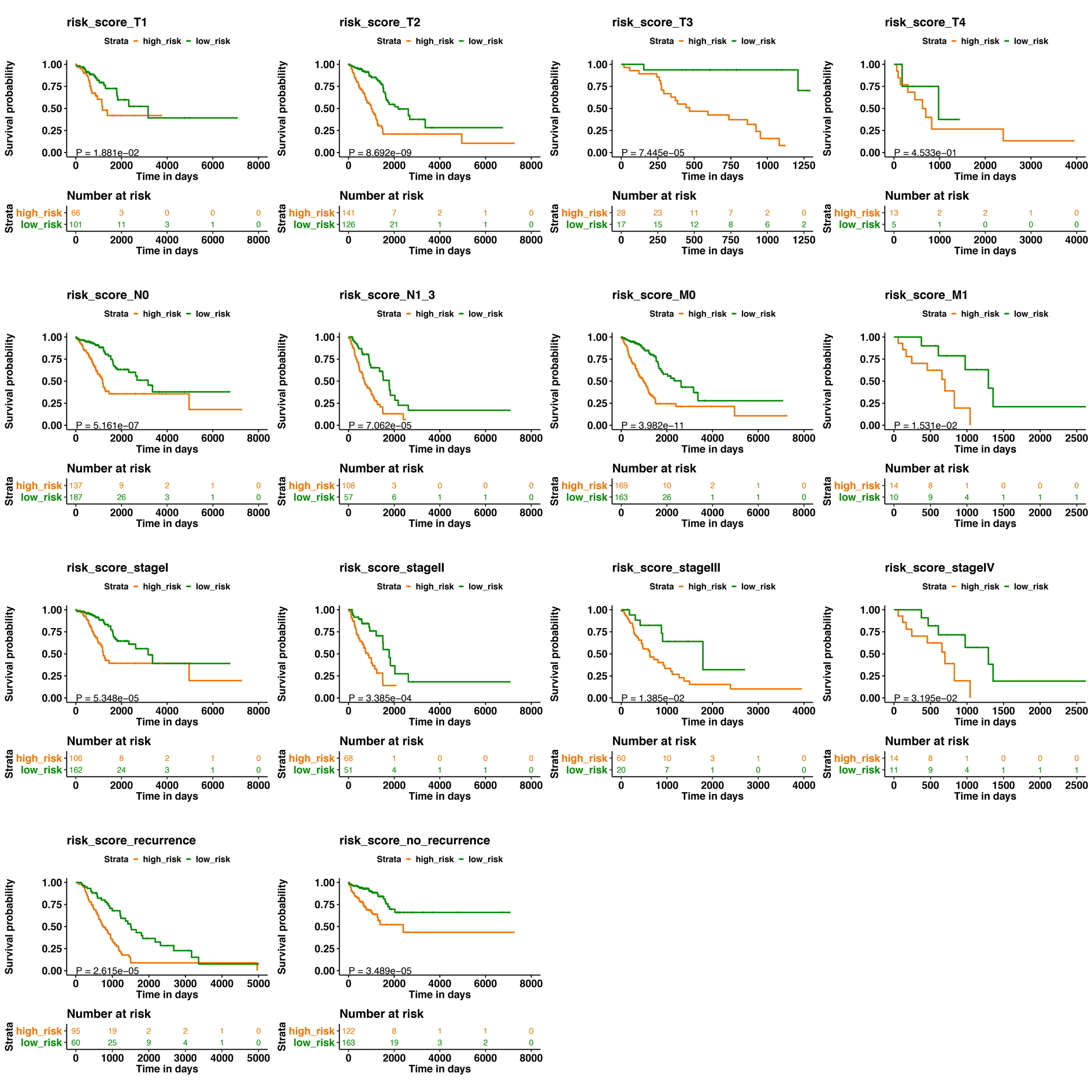

Supplement: Supplementary file 8 — Additional file 8: Figure S6. The Kaplan–Meier survival analysis of the signature for LUAD subgroup patients in TCGA dataset. Patients of high-risk exhibited poor prognosis in T1 stage cohort, T2 stage cohort, T3 stage cohort, N0 stage cohort, N1–3 stage cohort, M0 stage cohort, M1 stage cohort, stage I cohort, stage II cohort, stage III cohort, stage IV cohort, recurrence cohort, and no recurrence cohort (P < 0.05). There was no association of the risk score with patients of T4 stage cohort. Abbreviations: The Cancer Genome Atlas (TCGA). [file 12967_2019_1824_MOESM8_ESM.tif]

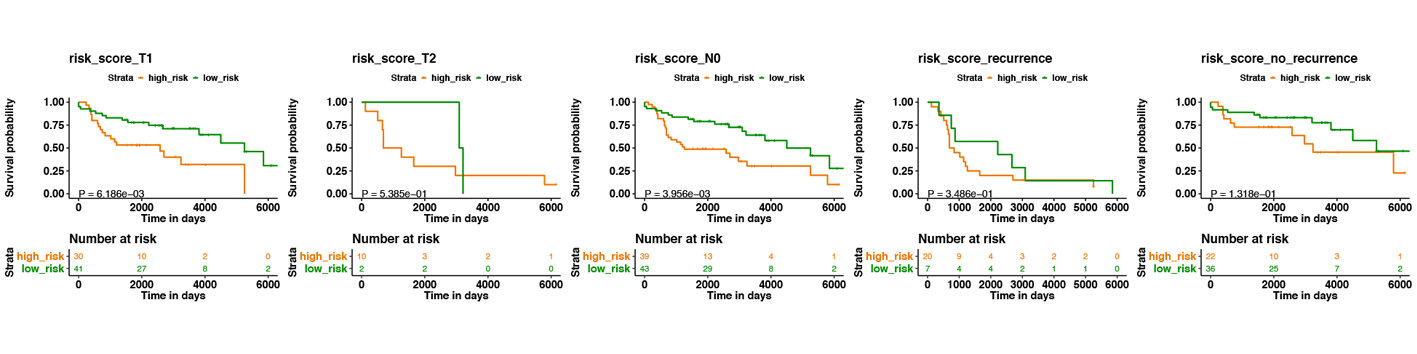

Supplement: Supplementary file 9 — Additional file 9: Figure S7. The Kaplan–Meier survival analysis of the signature for LUAD subgroup patients in GSE30219 dataset. Patients of high-risk exhibited poor prognosis in T1 stage cohort and N0 stage cohort (P < 0.05). There was no association of the risk score with patients of T2 stage cohort, recurrence cohort, and no recurrence cohort. [file 12967_2019_1824_MOESM9_ESM.tif]

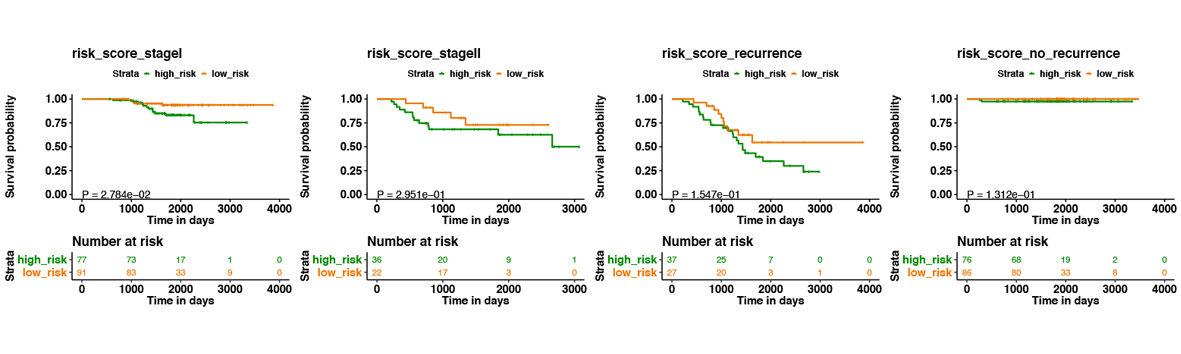

Supplement: Supplementary file 10 — Additional file 10: Figure S8. The Kaplan–Meier survival analysis of the signature for LUAD subgroup patients in GSE31210 dataset. Patients of high-risk exhibited poor prognosis in stage I cohort (P < 0.05). There was no association of the risk score with patients of stage II cohort, recurrence cohort, and no recurrence cohort. [file 12967_2019_1824_MOESM10_ESM.tif]

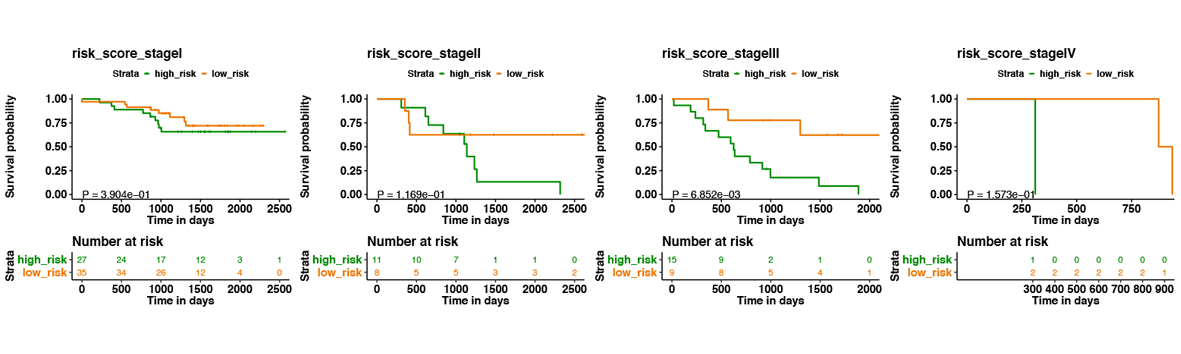

Supplement: Supplementary file 11 — Additional file 11: Figure S9. The Kaplan–Meier survival analysis of the signature for LUAD subgroup patients in GSE81089 dataset. Patients of high-risk exhibited poor prognosis in stage III cohort (P < 0.05). There was no association of the risk score with patients of stage I cohort, stage II cohort, and stage IV cohort. [file 12967_2019_1824_MOESM11_ESM.tif]

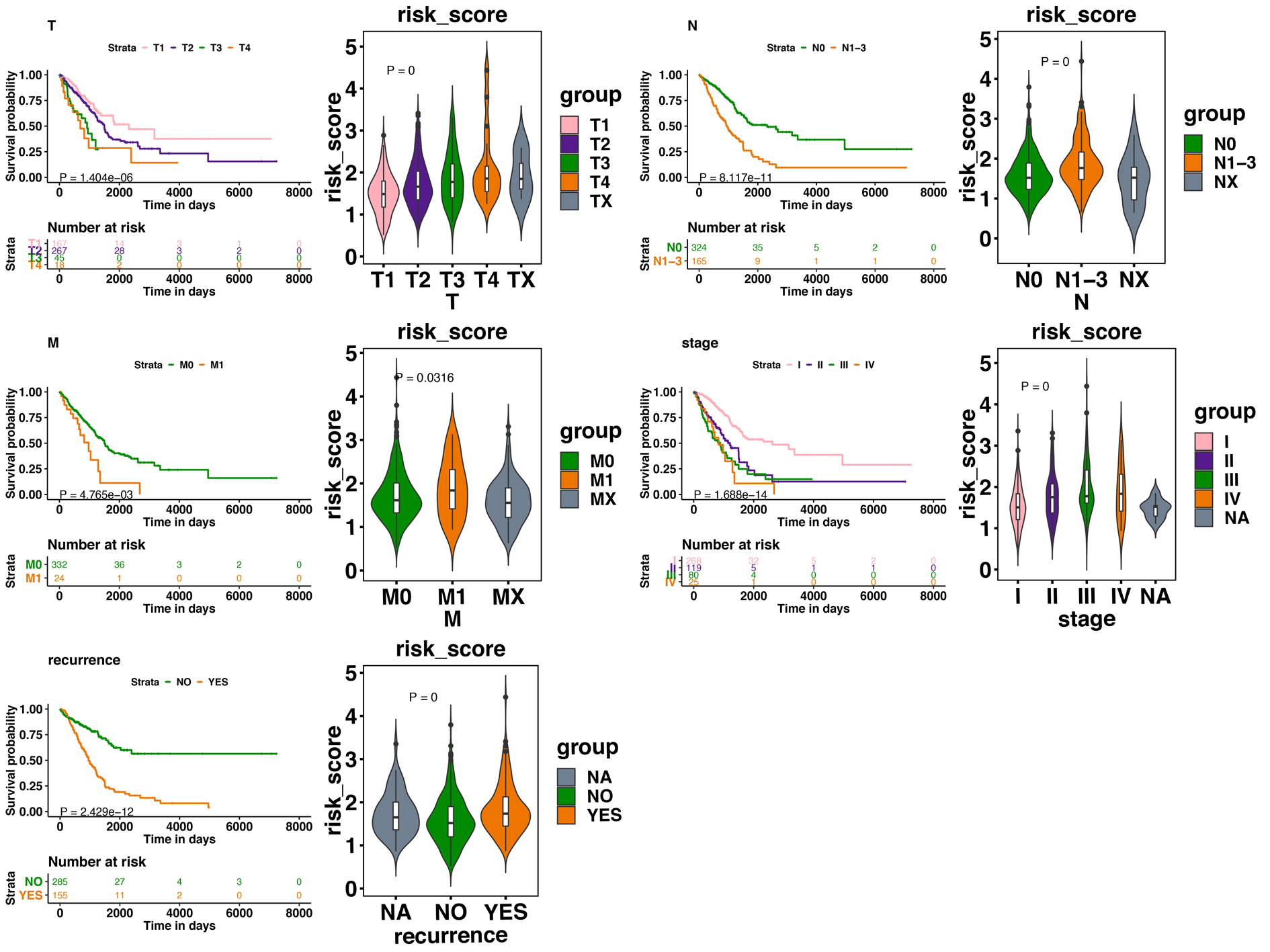

Supplement: Supplementary file 12 — Additional file 12: Figure S10. Correlation of the risk signature with clinicopathologic factors in TCGA datasets. The signature was positively correlated with T stage, N stage, M stage and pathologic stage in TCGA datasets (P < 0.05). Abbreviations: The Cancer Genome Atlas (TCGA). [file 12967_2019_1824_MOESM12_ESM.tif]

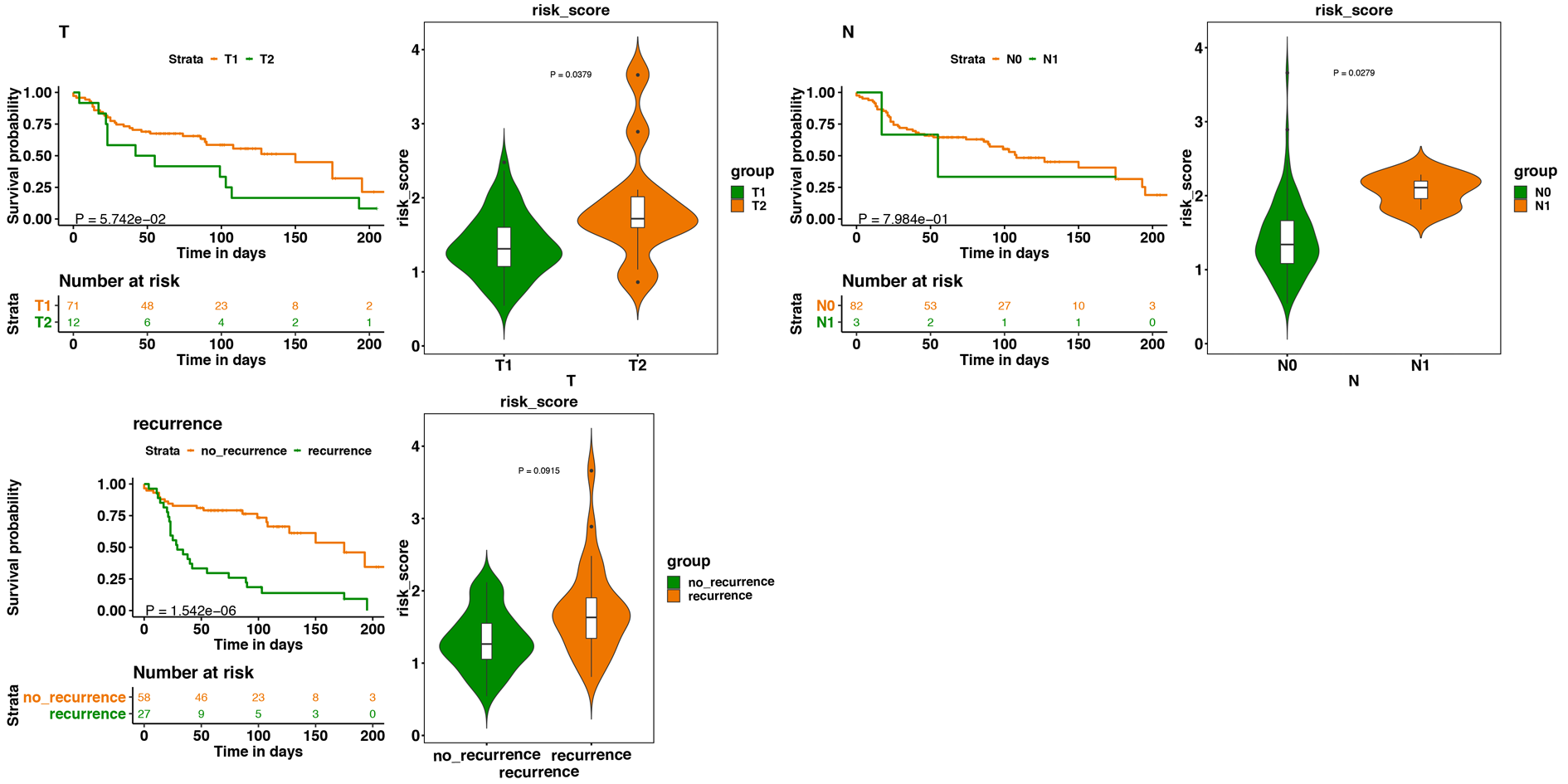

Supplement: Supplementary file 13 — Additional file 13: Figure S11. Correlation of the risk signature with clinicopathologic factors in GSE30219 datasets. The signature was positively correlated with T stage and N stage in GSE30219 datasets (P < 0.05). But there was no correlation of the signature and recurrence. [file 12967_2019_1824_MOESM13_ESM.tif]

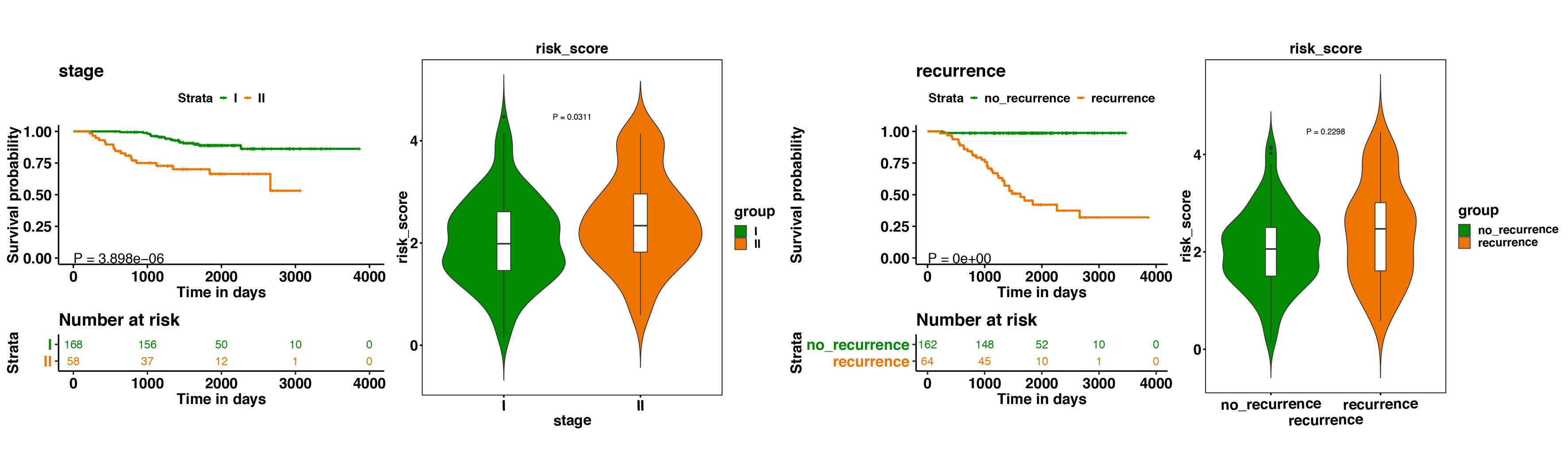

Supplement: Supplementary file 14 — Additional file 14: Figure S12. Correlation of the risk signature with clinicopathologic factors in GSE31210 datasets. The signature was positively correlated with pathologic stage in GSE31210 datasets (P < 0.05). But there was no correlation of the signature and recurrence. [file 12967_2019_1824_MOESM14_ESM.tif]

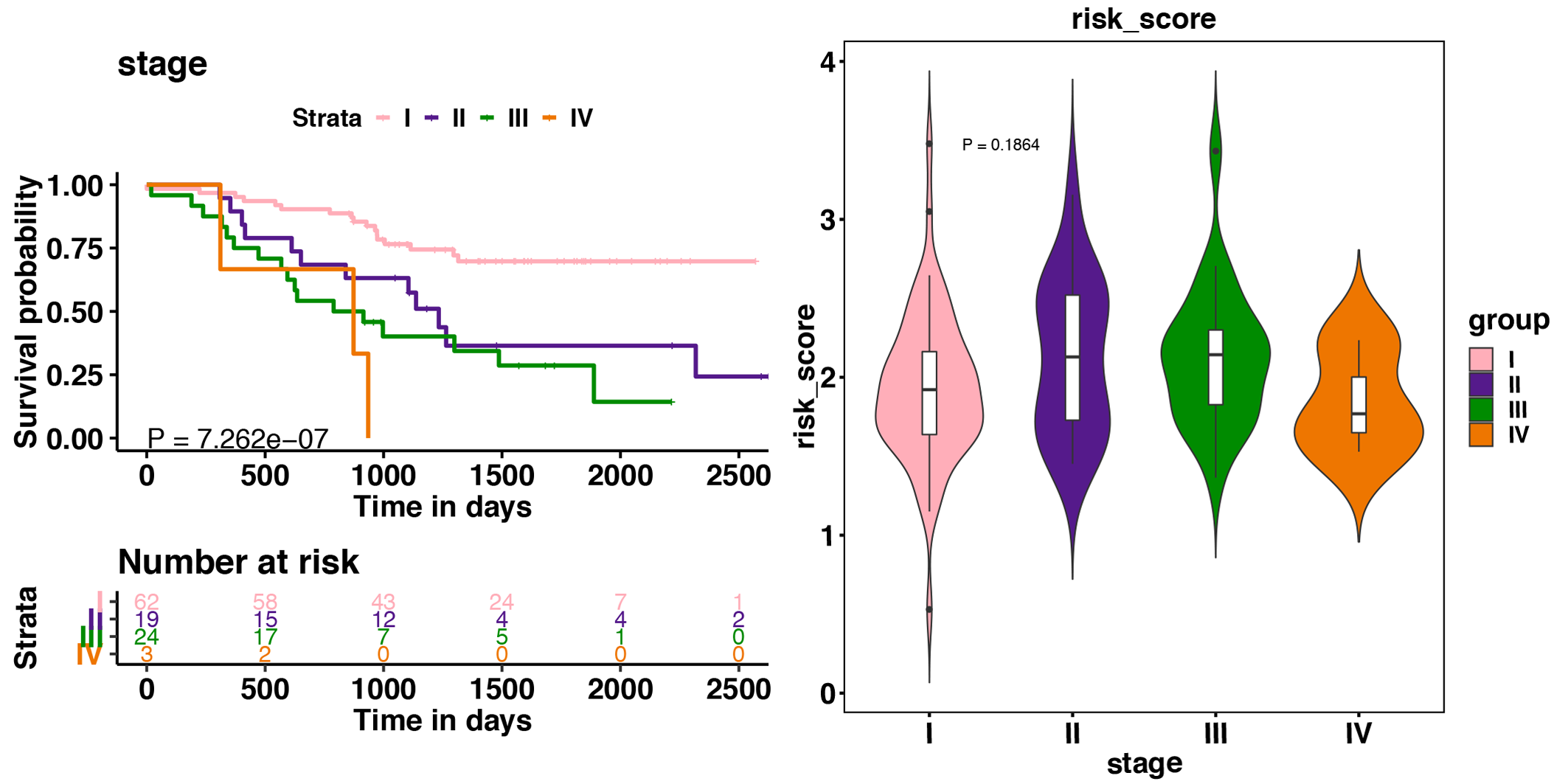

Supplement: Supplementary file 15 — Additional file 15: Figure S13. Correlation of the risk signature with clinicopathologic factors in GSE81089 datasets. There was no correlation of the signature and pathologic stage in GSE81089, which may be caused by the small number of stage IV patients. [file 12967_2019_1824_MOESM15_ESM.tif]
